# Bridging the scalability gap in van der Waals light guiding with high refractive index MoTe_2_

**DOI:** 10.1515/nanoph-2025-0468

**Published:** 2025-12-08

**Authors:** Mikhail K. Tatmyshevskiy, Georgy A. Ermolaev, Dmitriy V. Grudinin, Aleksandr S. Slavich, Nikolay V. Pak, Marwa A. El-Sayed, Alexander Melentev, Elena Zhukova, Roman I. Romanov, Dmitry I. Yakubovsky, Andrey A. Vyshnevyy, Sergey M. Novikov, Aleksey V. Arsenin, Valentyn S. Volkov

**Affiliations:** Moscow Center for Advanced Studies, 20 Kulakova str., Moscow, 141700, Russia; Emerging Technologies Research Center, XPANCEO, Internet City, Emmay Tower, Dubai, United Arab Emirates; Department of Physics, Faculty of Science, Menoufia University, Shebin El-Koom 32511, Egypt; Moscow Engineering Physics Institute, National Research Nuclear University MEPhI, 31 Kashirskoe Sh., 115409 Moscow, Russia

**Keywords:** high refractive index, all-dielectric nanophotonics, subwavelength waveguides, van der Waals

## Abstract

van der Waals transition metal dichalcogenides, distinguished by a high refractive index and giant optical anisotropy, are promising materials for integrated photonic devices. However, their superior optical properties are nowadays limited to exfoliated samples with only a micrometer scale, whereas industrial integration requires at least cm-scale dimensions. Here, we resolve this problem for MoTe_2_ by demonstrating that chemical vapor deposition synthesis can provide an identical optical response to the benchmark exfoliated samples in a broad spectral range (250–5,000 nm). It allows us to show high-performance waveguiding properties of MoTe_2_ with a subwavelength footprint of ∼*λ*/8 for telecommunication wavelengths. Therefore, our findings reveal MoTe_2_ as an ideal platform for the next-generation nanophotonics.

## 1 Introduction

The goal of miniaturizing integrated photonics to the level of electronics has been pursued for decades [1], [2], [3]. With the potential to overcome the diffraction limit, the most recent attempts used plasmonic nanostructures that support surface plasmon polaritons [4], [5], [6]. However, this approach faces a significant setback due to high ohmic losses, which limit the applications of plasmonic components and prevent the creation of chip-scale plasmonic integrated circuits [5], [7]. This highlights the urgent need for alternative lossless materials with exceptionally high refractive index *n* to overcome the limitations of the plasmonic approach [8], [9], [10].

**Figure 1:**
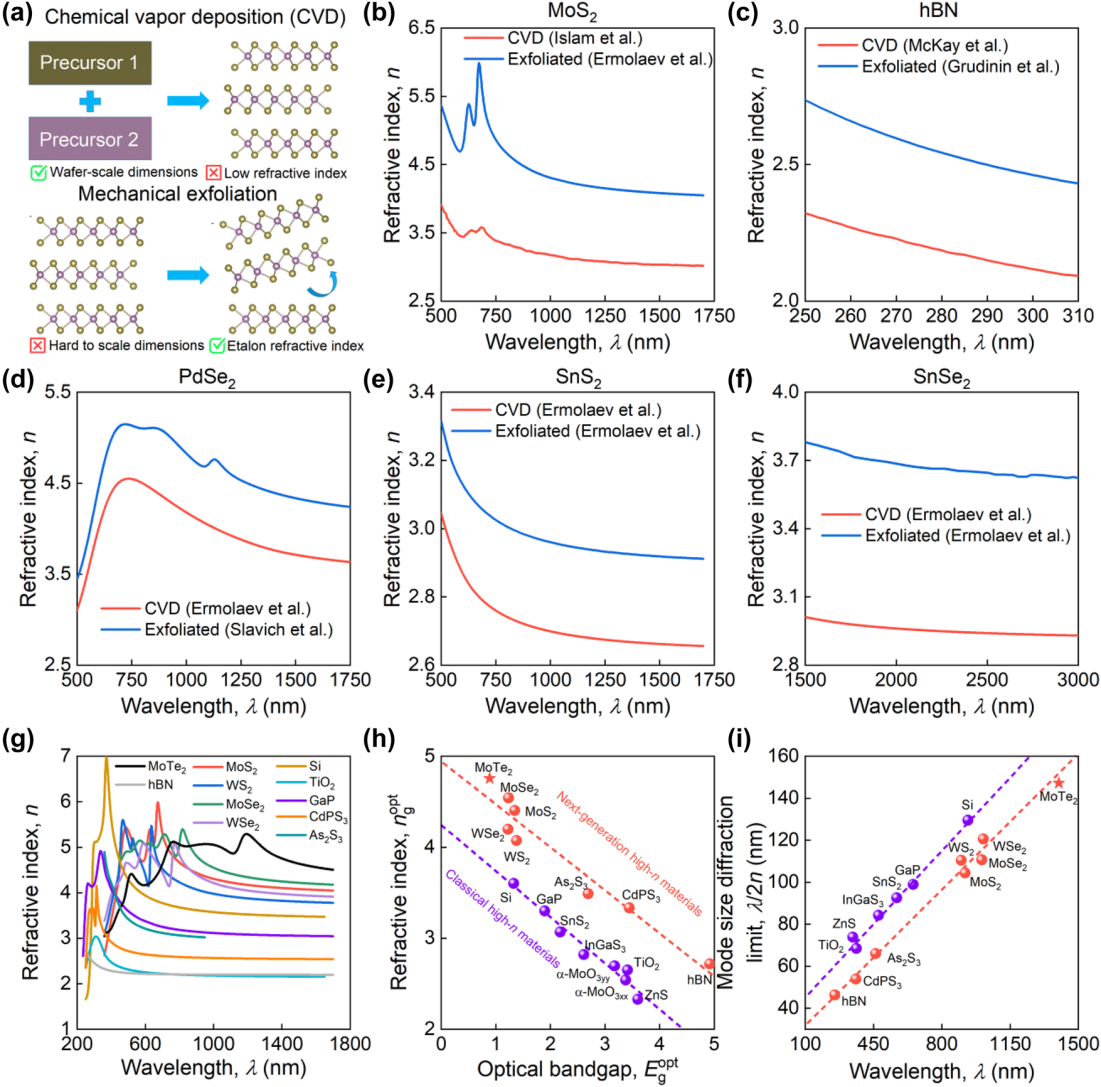
Refractive index of vdW materials. (a) Schematics of CVD and mechanical exfoliation fabrication methods of vdW materials. Comparison of the CVD and exfoliated refractive index for (b) MoS_2_, (c) hBN, (d) PdSe_2_, (e) SnS_2_, and (f) SnSe_2_. Optical constants are adopted from several reports [8], [10], [37], [38]. (g) Comparison of the refractive index of vdW materials with other high refractive index materials. Optical constants are adopted from the RefractiveIndex.Info database [39]. (h) Comparison of the maximum refractive index of vdW materials with other established highly refractive materials. (i) Comparison of the mode size, determined by diffraction limit *λ*/2*n*, for vdW materials and other established highly refractive materials. The dashed lines in panels (h) and (i) are the linear fitting of the data to demonstrate the linear correlations.

This challenge establishes a new branch of all-dielectric and integrated nanophotonics focusing on high-*n* materials [11], [12], [13], [14]. Further studies [15], [16], [17], [18], [19], [20], [21], [22], [23] reveal that van der Waals (vdW) materials, in particular, transition metal dichalcogenides (TMDCs) [24], [25], are ideal platforms for all-dielectric nanophotonics thanks to their record values of refractive index *n* ∼ 4 and pronounced excitons. However, these records are traditionally demonstrated for exfoliated samples, which are unsuitable for mass production because of their limited area with dimensions of only ∼10–100 µm [8], [26], [27]. On the other hand, alternative methods of large-scale vdW synthesis, such as molecular beam epitaxy (MBE) and chemical vapor deposition (CVD), have another problem: the refractive index of synthesized films is significantly lower than that of their exfoliated counterparts [28], [29], [30], or the refractive index does not change, but extinction appears [31], [32]. One might expect that in the transparency region, the refractive index is less sensitive to sample-specific variations. In reality, however, CVD-grown samples demonstrate a much lower refractive index compared to the etalon refractive index of exfoliated samples, as seen in Figure 1a–g. This difference originates from the fact that defects diminish materials’ resonances, such as excitons, oscillator strength, which significantly influences the refractive index due to Kramers–Kronig relations. At the same time, exfoliated vdW materials demonstrate the record-high refractive index in the transparency region, overcoming even the classical high-*n* materials, including Si, GaP, and TiO_2_ (Figures 1g and h). In Figure 1h, we plot this trade-off between transparency and refractive index for a range of materials. It is important to note that Figure 1h is designed as a materials-selection chart for nanophotonic applications. Therefore, the *x*-axis, labeled “Optical bandgap, $E_{g}^{\text{opt}}$ ,” represents a practical “transparency edge” rather than the fundamental quasiparticle bandgap. We define this edge as the photon energy at which the material’s extinction coefficient, *k*, exceeds a threshold of 0.001, corresponding to a material absorption loss of approximately 350 dB/cm at telecommunication wavelengths and also the limit of detection for traditional spectroscopic techniques like spectroscopic ellipsometry. This metric reflects the practical limit of useful transparency for compact on-chip devices. The *y*-axis (“Refractive index, $n_{g}^{\text{opt}}$ ”) shows the refractive index of each material measured at its respective transparency edge. This approach explains, for example, why silicon with fundamental bandgap of ∼1.1 eV is plotted at ∼1.33 eV [33], [34], the energy at which its weak indirect absorption surpasses our defined threshold of *k* = 0.001. In contrast, for van der Waals materials like hBN with strong excitonic features, the optical response is dominated by these resonances, which define the practical absorption limit at energies below the quasiparticle bandgap (∼5.9 eV) [35]. Meanwhile, the value of ∼4.9 eV in Figure 1h reflects the practical transparency edge of hBN [10], [36]. Still, from Figure 1h, we can clearly see that van der Waals materials show a higher refractive index in a given transparency region than classical high-refractive index materials. As a result, vdW materials offer a better mode size diffraction limit *λ*/2*n*, where *λ* is the wavelength and *n* is the refractive index, owing to the superior trade-off between optical bandgap and refractive index (Figure 1g). Therefore, finding vdW materials for which CVD or MBE films exhibit similar optical responses to exfoliated samples is in high demand.

In this work, we demonstrate that MoTe_2_ exhibits a unique combination of qualities, including a slight difference (of less than 1 % in the transparency range) between CVD MoTe_2_ film and exfoliated MoTe_2_ optical properties alongside the highest refractive index among all known TMDCs (MoS_2_, WS_2_, MoSe_2_, and WSe_2_) and transparency in the near-infrared region, as measured using broadband spectroscopic ellipsometry. Furthermore, we demonstrate that MoTe_2_ exhibits the best light-guiding properties with the footprint of ∼*λ*/8 using near-field optical microscopy. All these properties make MoTe_2_ a technological material for postsilicon nanophotonics.

## 2 Results

**Figure 2:**
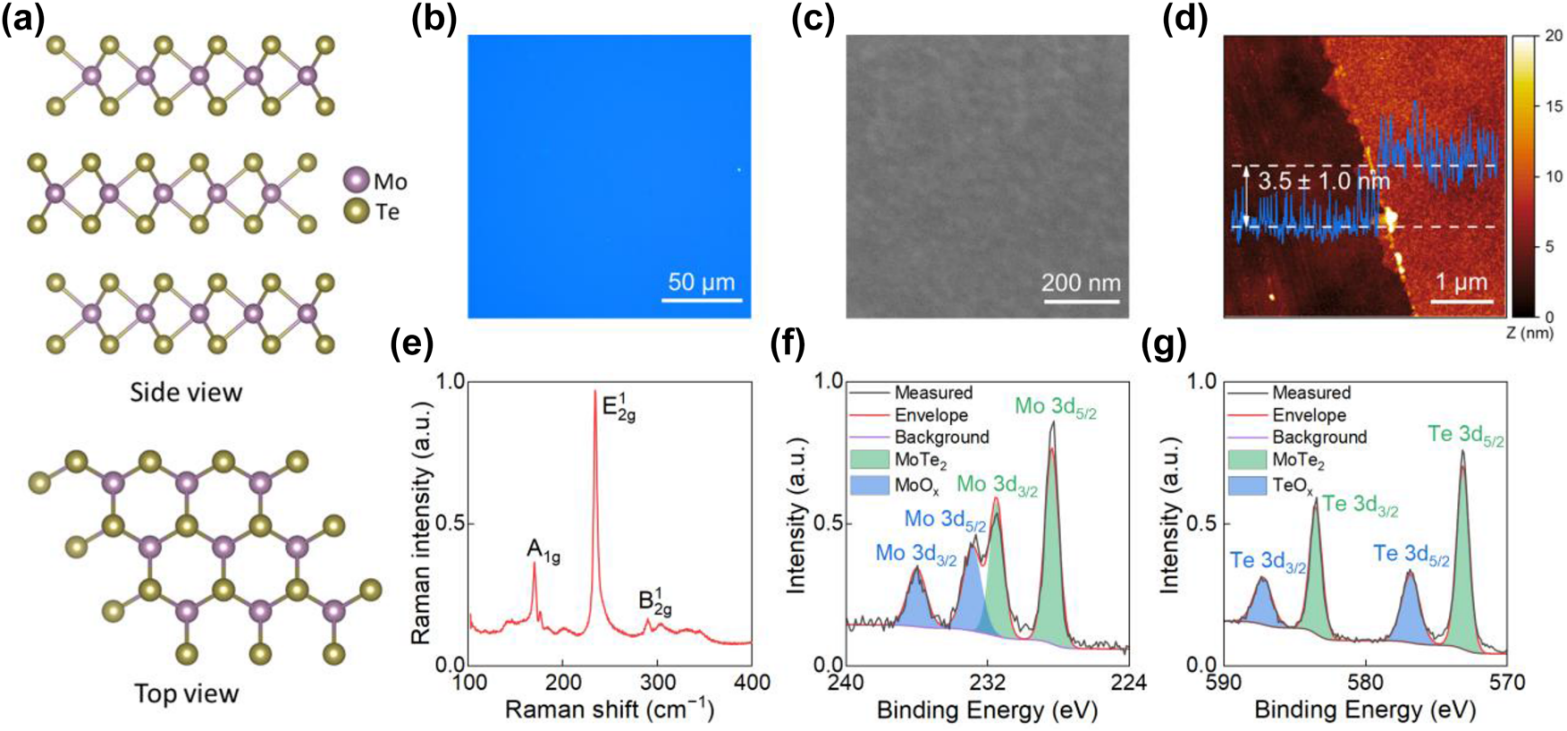
Structural and morphological characterization of MoTe_2_ film. (a) Crystal lattice structure of 2H–MoTe_2_, (b) optical image, (c) SEM image, (d) AFM image and height profile, (e) Raman spectrum, (f and g) XPS spectra of CVD MoTe_2_ film.

Figure 2a shows the crystal structure of a commercially synthesized CVD-grown (see Methods in Supplementary Information) 2H–MoTe_2_. Optical and scanning electron microscopy images in Figures 2b and c confirm the uniformity of our CVD MoTe_2_ film. The purpose of these images is to provide a visual inspection of the MoTe_2_ film at both microscale and macroscale. In addition, we performed atomic-force microscopy measurements to determine the film thickness (Figure 2d). Figure 2d shows that the thickness of our CVD-grown MoTe_2_ is 3.5 nm. To confirm the film’s crystal phase and chemical composition, we applied Raman spectroscopy and X-ray photoelectron spectroscopy (XPS). The Raman spectrum (Figure 2e) shows that our film has a prominent peak at about 234 cm^−1^, corresponding to $E_{2g}^{1}$ in-plane vibrational modes, and two less intensive peaks corresponding to *A*_1*g*_ (170 cm^−1^) and $B_{2g}^{1}$ (290 cm^−1^) out-of-plane vibrational modes, respectively. These peak positions agree with previously reported Raman spectra of MoTe_2_ [40]. Figures 2f and g shows the XPS spectra of Mo3d and Te3d core levels, respectively. The dominant doublets with 3d_5/2_ peaks positions at 228.4 eV and 573.2 eV are associated with the MoTe_2_ compound [41]. In addition, XPS spectra show the presence of MoO_x_ and TeO_x_ signals (the doublets with higher energies represent Mo and Te oxidized state [42], [43]), indicating surface oxidation (Figures 2f and g). Surface oxidation is a common phenomenon for many TMDCs, including MoTe_2_, when exposed to ambient conditions [44]. Hence, the Mo and Te oxidized states do not show the quality of the film. In contrast, the calculated Mo/Te ratio confirms the stoichiometry of our MoTe_2_ sample of a 33.5:66.5 ratio, which is very close to the expected 1:2 (see Supplementary Information).

**Figure 3:**
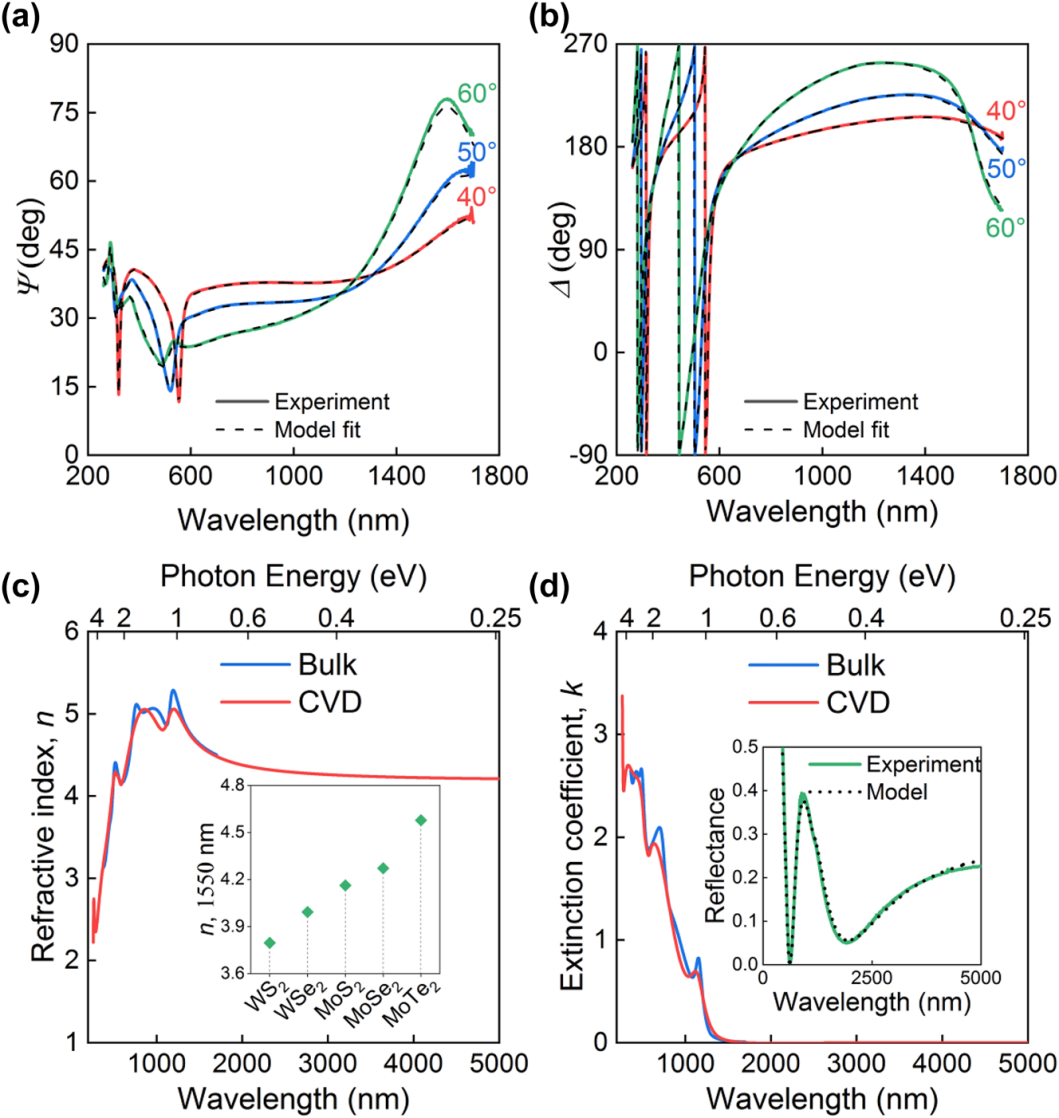
Ellipsometry measurements of MoTe_2_. (a,b) Experimental (solid lines) and calculated (dashed line) spectra of ellipsometric parameters Ψ and Δ, measured from MoTe_2_ thin film, (c) in-plane refractive index, and (d) in-plane extinction coefficients of bulk [50] and CVD (this work) MoTe_2_. Tabulated optical constants of MoTe_2_ are collected in Supplementary Information.

To determine the optical constants of our MoTe_2_ CVD film, we measure spectral ellipsometry and reflectance over a broad spectral range (*λ* = 250–1,700 nm for ellipsometry and *λ* = 450–5,000 nm for reflectance). The measured spectra for ellipsometry parameters Ψ and Δ are presented in Figures 3a and b, while the reflectance spectrum is shown in the inset of Figure 3d. In order to ensure consistency in the overlap range 450–1,700 nm of ellipsometry and reflectance, we use both spectra (from ellipsometry and reflectance) at the same time for fitting the optical properties of MoTe_2_. The model for processing ellipsometry data consists of four layers: the bottom 0.5 mm-thick layer of silicon, a layer of 286 nm SiO_2_, a *t*_MoTe2_ nm-thick MoTe_2_ layer, and a *t*_oxide_ nm-thick oxide layer, with the total thickness *t*_MoTe2_ + *t*_oxide_ = 3.5 nm as determined from the AFM profile (Figure 2d). The best-fit procedure yielded a thickness of *t*_MoTe2_ = 3.0 nm for the MoTe_2_ layer and *t*_oxide_ = 0.5 nm for the oxide. The minor influence of this sub-nanometer oxide on the extracted optical constants, confirmed by a comparative analysis in Supplementary Information, is a direct consequence of the overwhelming optical signature of the high-index MoTe_2_. In the optical thin regime (*t* ≪ *λ*), the contribution of a layer to the total reflection is approximately proportional to the product of its thickness, *t*, and its optical contrast [45], [46], [47], related to $n^{2}-n_{\text{env}}^{2}$ , where $n_{\text{env}}=\sqrt{\left(n_{\text{air}}^{2}+n_{\text{sub}}^{2}\right)/2}\approx 1.3$ (*n*_air_ = 1 and *n*_sub_ = *n*_SiO2_ ≈ 1.45) is the refractive index of the effective surrounding medium [48]. Given the large disparity in both thickness (*t*_MoTe2_ = 3.0 nm and *t*_oxide_ = 0.5 nm) and, more critically, refractive index (*n*_MoTe2_ ≈ 4.5 and *n*_oxide_ ≈ 2.0), the contribution of the MoTe_2_ layer dominates that of the oxide by a factor of $t_{\text{MoTe}2}\left(n_{\text{MoTe}2}^{2}-n_{\text{env}}^{2}\right)/t_{\text{oxide}}\left(n_{\text{oxide}}^{2}-n_{\text{env}}^{2}\right)\approx 50$ . Consequently, the fitting procedure is overwhelmingly sensitive to the optical properties of MoTe_2_, with the thin oxide representing only a minor perturbation to the overall optical response. For modeling the optical constants of MoTe_2_, we used the Tauc–Lorentz oscillators model, which has proven to describe the optical response of vdW materials extremely well [49]. The resulting in-plane refractive index *n* and extinction coefficient *k* of our CVD film are shown in Figures 3c and d. From Figures 3c and d, we notice that the optical constants of CVD MoTe_2_ almost coincide with the optical constants of exfoliated MoTe_2_ [50], especially in the telecommunication range (1,260–1,675 nm). Interestingly, neglecting an oxide layer in the optical model also gives similar optical properties for MoTe_2_ (see Supplementary Information). Additionally, we note that the refractive index of MoTe_2_ for these wavelengths is the largest among classical TMDCs (MoS_2_, WS_2_, MoSe_2_, and WSe_2_), as illustrated on the example of *λ* = 1,550 nm in the inset of Figure 3c.

Due to the atomic thickness of the film (3.0 nm) and the standard reflection-based ellipsometry configuration used, our measurements do not have sufficient sensitivity to reliably extract the out-of-plane optical constants *n*_c_ [51]. However, we can infer the expected anisotropy by referencing the recent comprehensive study on exfoliated MoTe_2_ [50]. Given that our CVD film shows an in-plane refractive index nearly identical to that of exfoliated samples, it is reasonable to expect that it possesses a similarly large optical anisotropy. Moreover, such optical anisotropy can be advantageous for certain nanophotonics applications, for example, optical anisotropy can expand the parameter space for achieving single-mode operation and can be used as a design parameter to optimize mode confinement and reduce crosstalk [9].

Certainly, this finding of equal refractive index of CVD-grown and exfoliated MoTe_2_ does not necessarily imply that all CVD-synthesized MoTe_2_ will exhibit identical optical constants to exfoliated material. The underlying physical mechanism enabling the “high-quality” of our specific CVD MoTe_2_ sample, in terms of matching optical constants to exfoliated samples, can be attributed to the thermodynamics of MoTe_2_ between 2H and 1T’ phases [40]. Unlike other TMDCs, MoTe_2_ has a relatively small energy difference (Δ*E* ≈ 45 meV) between its 2H (semiconducting) and 1T’ (metallic) phases [52]. Consequently, the 2H phase appears in CVD only within the very narrow synthesis parameters since even a small concentration of defects leads to the phase transition from 2H to 1T’. Therefore, the thermodynamics of MoTe_2_ ensures the high quality of 2H–MoTe_2_ because otherwise CVD results in 1T’-MoTe_2_, which is in stark contrast with other TMDCs like MoS_2_, where a much larger energy difference (Δ*E* ≈ 650 meV) [52] means that even a large concentration of defects cannot induce this phase transition. As a result, one can synthesize 2H–MoS_2_ under a wide range of conditions, leading to films with varying defect densities: a “bad” synthesis still yields a 2H film, albeit a low-quality one [28]. We believe it is one of the primary reasons why 2H–MoTe_2_ received considerably less attention compared to other TMDCs despite having the superior optical properties. However, the complexity of the 2H–MoTe_2_ synthesis results in low defect density films, once achieved. Since the 2H–MoTe_2_ and 1T’-MoTe_2_ phases are nearly degenerate in energy, this delicate balance is easily tipped by external factors, including strain [52], doping [53], and, most importantly, defects [40]. Therefore, the system effectively “refuses” to form a highly defective 2H–MoTe_2_ film [40]. Hence, the challenge of synthesizing 2H–MoTe_2_ is actually a great advantage for its practical applications, and this self-limiting growth kinetics of MoTe_2_ are the primary reason for reproducing the refractive index in both CVD-grown and exfoliated MoTe_2_.

From the optical constants in Figure 3, we can evaluate the defect concentration from optical absorption. Assuming a simplistic model of light absorbance in the sub-bandgap region being proportional to the density of defects [54], we can estimate, to the first order approximation, light absorbance *α* as *α* = *σN*_defects_, where *α* = 4*πk*/*λ* (*k* is the extinction coefficient), *σ* is the absorption cross section for a single defect site, and *N*_defects_ is the density of defect sites. The typical value of *σ* ranges from 10^−17^ to 10^−15^ cm^2^ [55]. Let us take the smallest value of *σ* = 10^−17^ cm^2^ to obtain the upper limit of *N*_defects_. For calculations, we take *λ* = 1,550 nm, which gives *N*_defects_ = 4*πk*/(*λσ*) ≈ 1.3·10^20^ atoms/cm^3^. To calculate the percentage of defects, we need the total density of atoms in MoTe_2_. 2H–MoTe_2_ has a hexagonal crystal structure with two formula units (MoTe_2_) per unit cell. The dimensions of the unit cell are *a* = 0.352 nm and *c* = 1.391 nm [56]. Using these values, the total density of atoms in MoTe_2_ is given by *N*_total_ = (atoms in unit cell)/*V*_unit cell_ = 6/(( $\sqrt{3}$ /2)*a* ^2^ *c*) ≈ 4·10^22^ atoms/cm^3^. Therefore, the upper boundary of defects percentage in our sample is given by *N*_defects_/*N*_total_ ≈ 0.3 %, which is an order of magnitude less than 3 % required for the phase transition from 2H to 1T’. This value is also in agreement with XPS data that, indicating the stoichiometry of our MoTe_2_ is 33.5:66.5, as (33.5–0.1 %):(66.5 + 0.2 %) ≈ 0.502, which is within 0.4 % accuracy of the ideal stoichiometry ratio of 0.5. These defects’ optical losses have a negligible impact on waveguide mode propagation direction, scattering losses, and confinement in planar waveguides since they are determined mostly by the refractive index.

**Figure 4:**
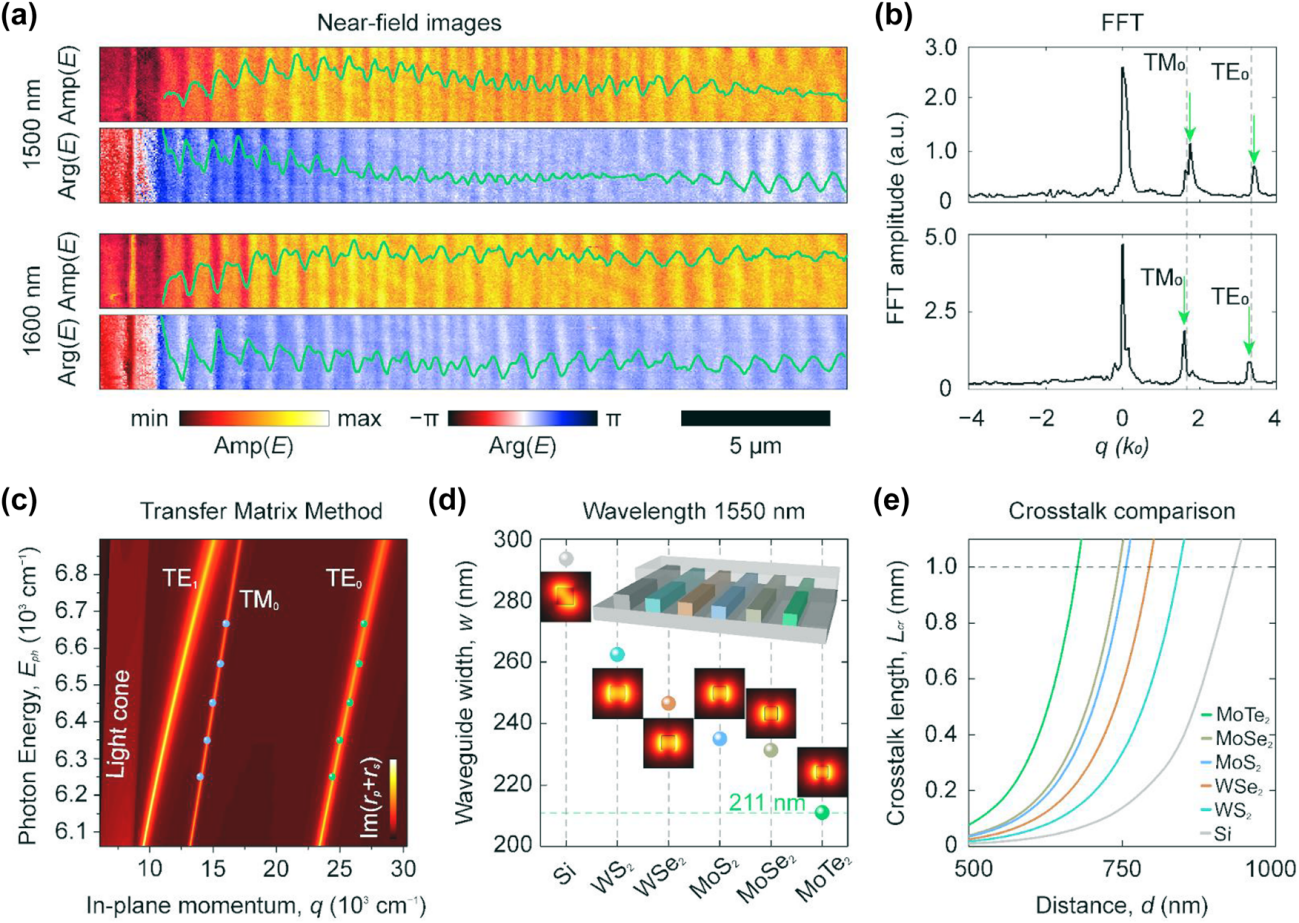
Near-field study of MoTe_2_ planar waveguides. (a) Near-field images, amplitude Amp(*E*), and phase Arg(*E*) for 1,500 and 1,600 nm. Other wavelengths are presented in Supplementary Information. (b) Fast Fourier Transform (FFT) of the complex near-field signal from panel (a). Green arrows mark the peak associated with the waveguide modes. (c) Transfer matrix calculations for planar MoTe_2_ waveguide. The experimental (*q* = 1/*λ*, *E* = *hc*/*λ*) data points (blue and green circles) show good agreement with the calculated dispersion. (d) Comparison of waveguide widths derived from multiple materials, each optimized to achieve the same effective mode index in the fundamental mode. The waveguides were fully encapsulated in SiO_2_ and had square cross sections. Their widths were optimized so that the fundamental mode would have the same effective refractive index. The inset shows a schematic image of the encapsulated waveguides. (e) Relation of crosstalk length and distance between cores of the waveguides for high-refractive index materials at 1,550 nm wavelength. The width is optimized for each point.

To demonstrate the waveguiding properties of MoTe_2_, we study planar MoTe_2_ waveguides using scattering near-field optical microscopy (s-SNOM). The s-SNOM measurements were performed on a thicker (≈235 nm) MoTe_2_ flake prepared by mechanical exfoliation. The purpose of this experiment was to demonstrate the excellent waveguiding properties stemming from MoTe_2_ intrinsic high refractive index. For the measurements, we used a standard telecommunication wavelength range of 1,500–1,600 nm to excite the waveguiding mode inside the MoTe_2_ planar waveguide by focusing the light on the tip of an s-SNOM. Excited mode travels inside the waveguide and scatters on the edge. That scattering field interferes with the background signal, thus giving us the oscillating pattern (Figure 4a). To retrieve guiding mode properties, we studied their fast Fourier transform (Figure 4b). It requires taking into account the frequency shift [57]:

$$n_{\text{eff}}=n_{\text{obs}}-\cos\alpha \sin\beta ,$$

where *n*_eff_ is the effective mode index, *n*_obs_ is the observed effective mode index, *α* is the angle between the illumination wavevector *k* and its projection on the sample surface *k*_‖_, and *β* is the angle between *k*_‖_ and the edge of the planar waveguide, where scattering occurs. Given the extracted refractive index components *n*_*ab*_ and frequency shift, we calculate the energy (*E* = *hc*/*λ*)-momentum (*q* = 1/*λ*) dispersion relation of the waveguide mode using the transfer matrix method [58] (see Supplementary Information). Plotting experimental effective indices on this map (Figure 4c) shows excellent agreement between experiment and theory.

To assess MoTe_2_ as a high refractive index photonic material, we compare MoTe_2_ waveguiding properties with other high refractive vdW materials, including MoS_2_, WS_2_, MoSe_2_, and WSe_2_. We consider waveguides operating at standard telecommunication wavelength *λ* = 1,550 nm. To enable a fair comparison, the waveguide widths were individually optimized so that the fundamental mode in each case exhibited the same effective refractive index. Therefore, although the physical dimensions vary, the mode confinement condition was held constant across all materials. For material analysis, we found the widths of square-shaped waveguides encapsulated in SiO_2_ with a given exponential decay *χ* = 0.89 μm^−1^ of the evanescent tail of the mode field outside the waveguide core, defined as:

$$\chi =\frac{2\pi }{\lambda }\sqrt{n_{\text{eff}}^{2}-n_{\text{Si}{\text{O}}_{2}}^{2}}.$$

Figure 4d confirms that MoTe_2_ waveguide core is the smallest compared to Si and other TMDCs. The results of waveguide crosstalk studies ( $L_{\text{ct}}=\lambda /\left(2\left|n_{o}-n_{e}\right|\right)$ , with *n*_o_ and *n*_e_ being effective indices of odd and even supermodes of a pair of parallel waveguides) in Figure 4e show that MoTe_2_ demonstrates the highest integration density.

## 3 Conclusions

In summary, we have reported the broadband (250–5,000 nm) optical constants of CVD MoTe_2_ and shown that its refractive index is in good agreement with the refractive index of exfoliated MoTe_2_, opening an avenue for industrial-scale use of MoTe_2_ in photonics. Moreover, MoTe_2_ transparency and a high refractive index of ∼4.5 in the near-infrared region result in high-performance waveguides with a footprint of ∼*λ*/8. Combined with the fact that CVD MoTe_2_ is like exfoliated MoTe_2_ in terms of optical properties, this material is a promising platform for nanophotonics.

Looking forward, the availability of scalable, high-quality MoTe_2_ opens numerous avenues for advanced nanophotonics devices [26], such as δ-waveguides [59] (see Supplementary Information) and integrated nanophotonics [9]. Furthermore, high-index MoTe_2_ nanostructures are ideal candidates for realizing high-*Q* Mie-resonant metasurfaces [16] for applications in sensing [14], flat optics [60], and enhanced light–matter interactions [11]. Finally, as MoTe_2_ is known to host defect-based quantum emitters in the telecommunication spectral range [61], our work could establish a viable path toward integrating single-photon sources into high-index photonic circuits for scalable photonic quantum information technologies.

## Supplementary information

Supplementary Information contains sections Materials and Methods, Additional Figures, and tabulated optical constants.

## Supplementary Material

Supplementary Material Details
