# Supplementary material for: Bridging the scalability gap in van der Waals light guiding with high refractive index MoTe2
Source: Nanophotonics. 2025 Dec 8;14(27):5427–35. doi: 10.1515/nanoph-2025-0468 (PMC12717896; doi:10.1515/nanoph-2025-0468)
Supplement: Supplementary file 1 — Supplementary Material Details [file j_nanoph-2025-0468_suppl_001.docx]

**Supplementary Information for**

**Bridging the scalability gap in van der Waals light guiding with high refractive index MoTe_2_**

Mikhail K. Tatmyshevskiy^1†^, Georgy A. Ermolaev^2†*^, Dmitriy V. Grudinin^2†^, Aleksandr S. Slavich^2^, Nikolay V. Pak^1,2^, Marwa A. El-Sayed^1,3^, Alexander Melentev^1^, Elena Zhukova^1^, Roman I. Romanov^4^, Dmitry I. Yakubovsky^1^, Andrey A. Vyshnevyy^2^, Sergey M. Novikov^1^, Aleksey V. Arsenin^2^, Valentyn S. Volkov^2^

*^1^Moscow Center for Advanced Studies, 20 Kulakova str., Moscow, 141700, Russia*

*^2^Emerging Technologies Research Center, XPANCEO, Internet City, Emmay Tower, Dubai, United Arab Emirates*

*^3^Department of Physics, Faculty of Science, Menoufia University, Shebin El-Koom 32511, Egypt*

*^4^Moscow Engineering Physics Institute, National Research Nuclear University MEPhI, 31 Kashirskoe Sh., 115409 Moscow, Russia*

^†^These authors contributed equally to this work

**Correspondence should be addressed to the e-mail: ermolaev-georgy@xpanceo.com*

**Materials and Methods**

**Materials.** CVD-grown full-coverage multi-layer MoTe_2_ samples were purchased from 2d Semiconductors, Inc. (2d Semiconductors Inc., Scottsdale, AZ, USA). The films were grown on c-cut sapphire substrates using the low-pressure chemical vapor deposition method (LPCVD).

**Raman Spectroscopy.** Raman spectra of MoTe_2_ films were measured using a Horiba LabRAM HR Evolution confocal Raman microscope (HORIBA Ltd., Kyoto, Japan), equipped with a 632.8 nm helium-neon laser, 1800 lines/mm diffraction grating, and ×100 objective (N.A. = 0.90) in a spectral range of 100–400 cm^−1^. The Raman spectra were recorded with 0.5 mW power and an integration time of 20 s at each point. The statistics were collected from 10 points for each sample.

**X-ray Photoelectron Spectroscopy.** The films’ chemical state and composition were analyzed by XPS in the Theta Probe tool (Thermo Scientific) under high-vacuum conditions with a monochromatic Al-Kα X-ray source (1486.6 eV). Photoelectron spectra were acquired using fixed analyzer transmission (FAT) mode with 50 eV pass energy. XPS spectra were acquired using charge-compensation under a pressure of $\sim$10^-7^ mbar. The spectrometer energy scale was calibrated on the Au4f_7/2_ line (84.0 eV).

**Energy-dispersive X-ray spectroscopy.** The stoichiometry of the MoTe_2_ film was verified by an energy dispersive X-ray spectroscopy system (EDS, Bruker QUANTAX EDX) set in an electron microscope (SEM, JEOL JSM-7001F) working in secondary electron imaging mode. The value of the acceleration voltage, 7 keV, was chosen to minimize the influence of the Si peak on the determination of the atomic ratio of the elements. The EDS spectrum was analysed using specialized software provided by Bruker based on the ZAF corrections method.

**Atomic Force Microscopy.** AFM was utilized to characterize the surface topography and morphological features of the CVD MoTe₂ films under investigation. Measurements were conducted using an NT-MDT Spectrum Instruments NTEGRA II AFM in Hybrid mode under ambient conditions. The cantilevers employed were NSA01 tips (TipsNano), characterized by a spring constant of 5.1 N/m, a tip radius of less than 10 nm, and a resonant frequency of 150 kHz. Scanning was performed at a rate of 0.5 Hz to capture detailed images of the CVD MoTe2 film surfaces and steps. Subsequent AFM data analysis was carried out using Gwyddion software, facilitating a comprehensive examination of the film's structural properties.

**Optical Visualization.** The optical images of MoTe_2_ films were captured using an optical microscope (Nikon LV150L, Tokyo, Japan) with an ×50 objective and a digital camera DS-Fi3.

**Scanning Electron Microscopy.** Surface morphology and homogeneity of the MoTe_2_ film were visualized by a scanning electron microscope JEOL JSM-7001F (JEOL Ltd., Tokyo, Japan) with a Schottky emitter working in secondary electron imaging mode with a voltage of 30 keV and a working distance of 6.5 mm.

**Spectroscopic Ellipsometry.** Spectroscopic ellipsometry measurements were performed with a commercial imaging spectroscopic ellipsometer Accurion ep4 (Park Systems GmbH), equipped with a ×7 objective. Ellipsometric measurements were carried out in a wide wavelength range (250 – 1700 nm with 1 nm step) and three angles of incidence: 40°, 50° and 60°. All of the spectra were acquired in rotating compensator ellipsometry mode (RCE) from a $\sim$200 × 200 μm area of the sample. The ability to visualize a sample with high resolution allowed us to prove the uniformity of the ellipsometric data acquired from the sample area. The surface oxide layer, a standard feature for MoTe₂ exposed to ambient conditions, was accounted for in our optical model using an effective medium approximation, with optical constants derived from a linear combination of literature values for MoO₃ and TeO₂. The total thickness of the MoTe₂ and oxide layers was constrained to the 3.5 nm value determined by AFM.

**Reflection Spectroscopy.** The reflectance measurements in the visible range (450-900nm) were performed via an optical microscope RX50M equipped with a halogen light source. The reflected light was collected by an objective SOPTOP MPlanFL, ×20, N.A. 0.5 and directed to the grating spectrometer (Optosky ATP5020P) via an optical fiber (Thorlabs M92L02) using the setup described in work^[1]^. The infrared (900 nm - 5000 nm) parts of the reflectance spectra were measured using a Vertex 80v FTIR spectrometer with Hyperion 3000 microscope by Bruker. The microscope was equipped with a 15x reflective objective and an integrated aperture wheel. A Globar and a halogen lamp were used as light sources in the mid-infrared and near-infrared regions, respectively, and an MCT detector was used for both.

**Scanning Near-Field Optical Microscopy.** Near-field imaging was performed with the use of a commercially available scattering scanning near-field optical microscope (s-SNOM, neaSNOM www.neaspec.com). To excite waveguiding modes in the MoTe_2_ flake, we used a continuous wave Agilent 81600B tunable laser with a tunability range of 1500-1600 nm. As a scattering probe, we used a Pt/Ir-coated silicon tip with a resonance frequency of Ω ≈ 280 kHz and an oscillation amplitude of ~134 nm (ARROW-NCPt-50). The microscope operated in the reflection mode. To suppress the noise in the near-field images, we used an interferometric pseudoheterodyne approach at high harmonics, nΩ (in our case, third harmonics).

**Additional Figures and Information**


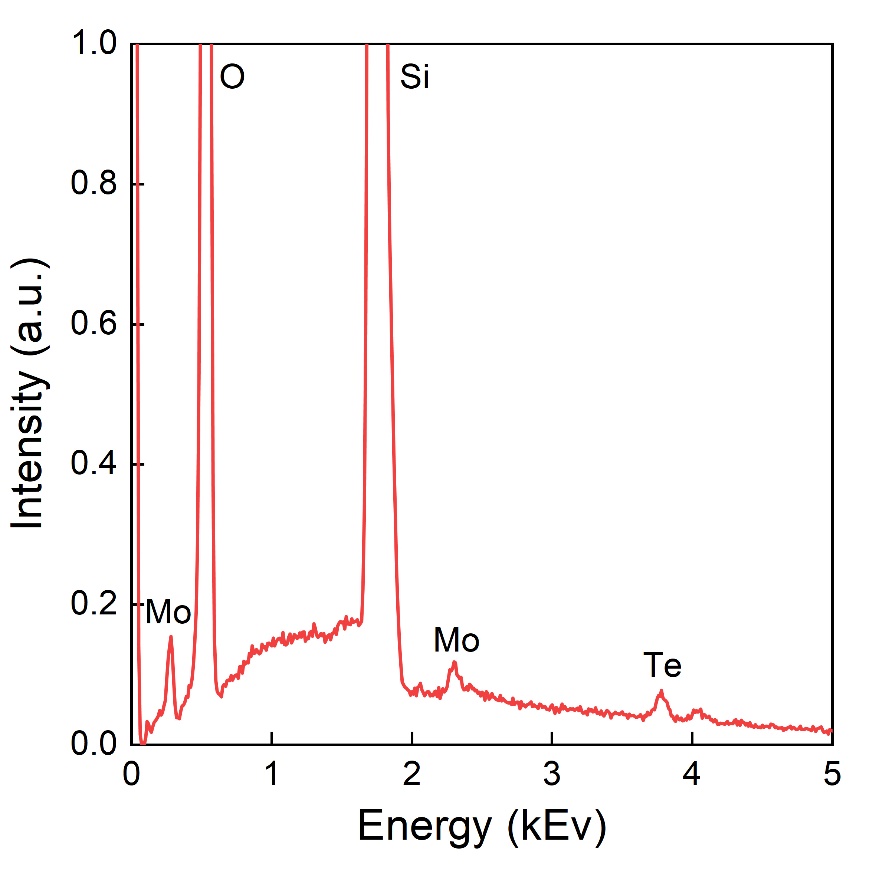


**Figure S1.** Energy dispersive spectroscopy of MoTe_2_ film. Stoichiometry of the sample, derived from the EDS spectrum, is 33.48:66.52, which is very close to the expected 1:2 ratio.


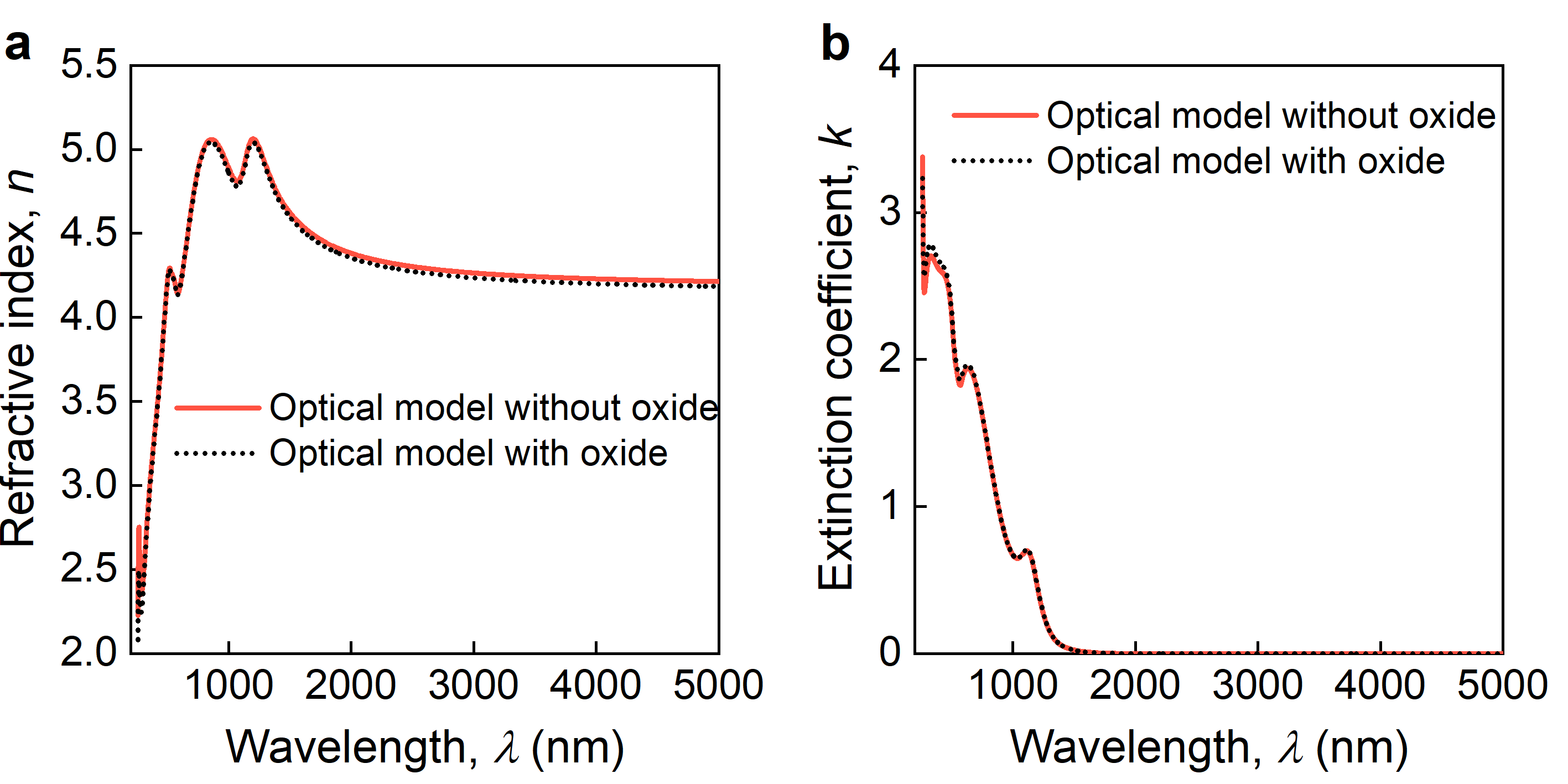


**Figure S2.** In-plane optical constants of MoTe_2_ obtained from fitting ellipsometry and reflectance spectra using two optical models with and without an oxide layer: (a) refractive index and (b) extinction coefficient.


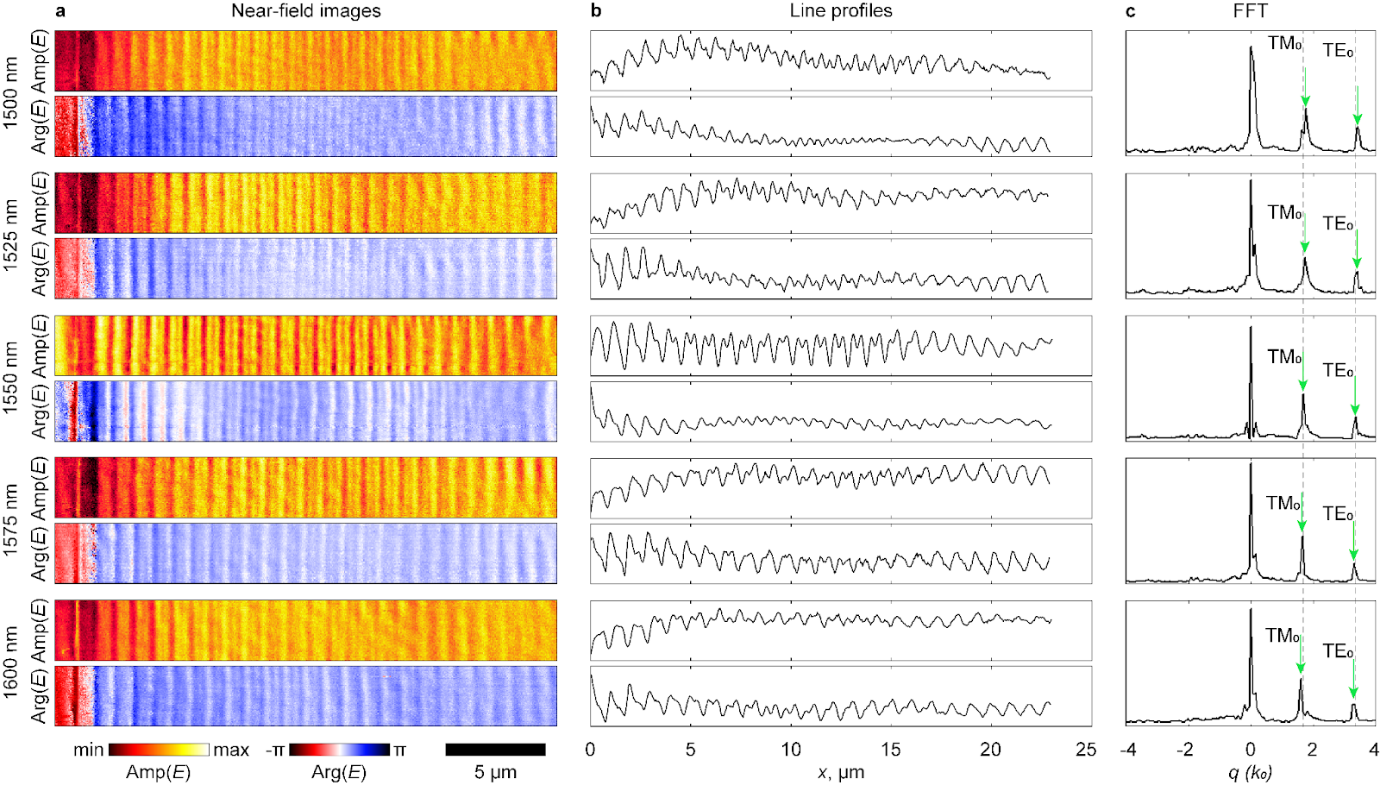


**Figure S3.** Near-field study of MoTe_2_ planar waveguides. (a) Near-field images, amplitude Amp(E) and phase Arg(E), (b) line profiles, derived from near-field images, (c) Fast Fourier Transform (FFT) of the complex near-field signal.


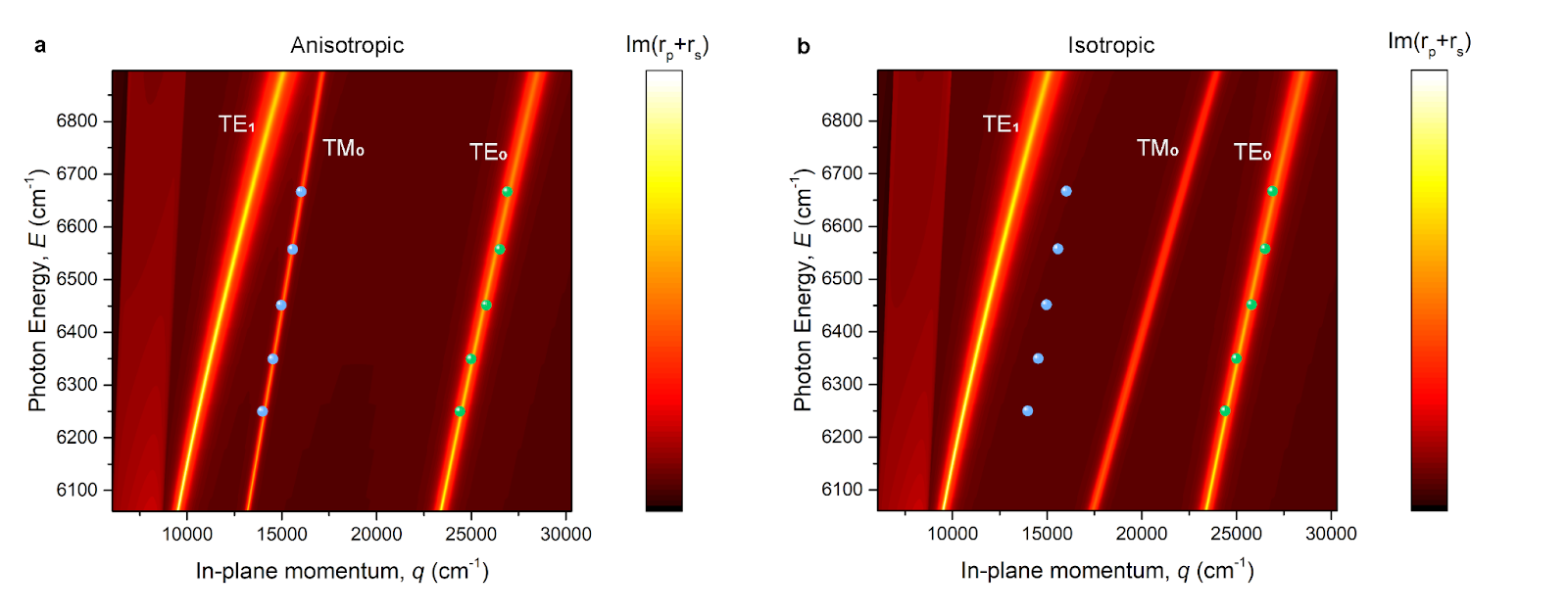


**Figure S4.** Transfer matrix calculations for a planar MoTe_2_ waveguide for (a) anisotropic and (b) isotropic optical models. The experimental (q = 1/λ, E = hc/λ) data points (blue and green circles) show good agreement with the anisotropic model calculations.

**Principles of transfer matrix calculations**

To calculate the transmission and reflection of plane waves for multilayered structures, it is convenient to use the transfer matrix method. The approach is based on the fact that two types of plane waves exist in each layer: plane waves propagating to the right (forward-propagating) and plane waves propagating to the left (backward propagating). These waves sum up into a collective plane waves propagating to the left. Determining the propagation of plane waves is equivalent to determining the amplitudes (*A* and *B*) for this part of waves in each layer. In the case of propagation within layer *i*, the relationship between the amplitudes across a distance *d*_i_ is described by the transfer matrix *P* (*z* is a coordinate perpendicular to the layers of the considered layered structure):

$\left( \begin{matrix} A\left( z+d \right) \\ B\left( z+d \right) \end{matrix} \right)=P_{i}\left( \begin{matrix} A\left( z \right) \\ B\left( z \right) \end{matrix} \right)=\left( \begin{matrix} e^{ik_{z,i}d_{i}} & 0 \\ 0 & e^{-ik_{z,i}d_{i}} \end{matrix} \right)$ (1)

, where $k_{z,i}=\sqrt{\varepsilon k_{0}^{2}-k_{x}^{2}}$ ($\varepsilon$ is the dielectric function of the layer, $k_{0}={2\pi}/{\lambda_{0}}$, where $\lambda_{0}$ is the wavelength of light in the vacuum, $k_{x}$ is the in-plane wave vector). Therefore:

$P_{i}=\left( \begin{matrix} e^{ik_{z,i}d_{i}} & 0 \\ 0 & e^{-ik_{z,i}d_{i}} \end{matrix} \right)$ (2)

In the case of the layer interface, the relationship between the amplitudes can be found using the Fresnel equations from the reflection and transmission coefficients for the boundary between two media. Then, the relationship between the amplitudes at the interface can be written as:

$\left\{ \begin{matrix} A\left( z+ \right)=t_{i-1,i}A\left( z- \right)+r_{i,i-1}B\left( z+ \right) \\ B\left( z- \right)=r_{i-1,i}A\left( z- \right)+t_{i-1,i}B\left( z+ \right) \end{matrix} \right.$ (3)

If we express $A\left( z+ \right)$ and $B\left( z+ \right)$ in terms of $A\left( z- \right)$ and $B\left( z- \right)$, we can find the transfer matrix $T_{i,i-1}$ for the interface between the layers:

$\left( \begin{matrix} A\left( z+ \right) \\ B\left( z+ \right) \end{matrix} \right)=T_{i,i-1}\left( \begin{matrix} A\left( z- \right) \\ B\left( z- \right) \end{matrix} \right)=\frac{1}{t_{i,i-1}}\left( \begin{matrix} t_{i-1,i}t_{i,i-1}-r_{i-1,i}r_{i,i-1} & r_{i,i-1} \\ -r_{i-1,i} & 1 \end{matrix} \right)\left( \begin{matrix} A\left( z- \right) \\ B\left( z- \right) \end{matrix} \right)$ (4)

Now, using the properties of the Fresnel coefficients: $-r_{i-1,i}=r_{i,i-1}$ and $t_{i-1,i}t_{i,i-1}-r_{i-1,i}r_{i,i-1}=1$. As a result, we obtain:

$T_{i,i-1}=\frac{1}{t_{i,i-1}}\left( \begin{matrix} 1 & r_{i,i-1} \\ -r_{i,i-1} & 1 \end{matrix} \right)$ (5)

As a result, the transfer matrix *M* for the entire multilayered structure can be written as:

$\left( \begin{matrix} A_{n} \\ B_{n} \end{matrix} \right)=M\left( \begin{matrix} A_{0} \\ B_{0} \end{matrix} \right)=T_{n,n-1}P_{n-1}\cdots T_{2,1}P_{1}T_{1,0}\left( \begin{matrix} A_{0} \\ B_{0} \end{matrix} \right)$ (6)

Meaning, the transfer matrix for the multilayer structure is:

$M=\frac{1}{t_{n,n-1}}\left( \begin{matrix} 1 & r_{n,n-1} \\ r_{n,n-1} & 1 \end{matrix} \right)\left( \begin{matrix} e^{ik_{z,n-1}d_{n-1}} & 0 \\ 0 & e^{-ik_{z,n-1}d_{n-1}} \end{matrix} \right)\cdots\frac{1}{t_{21}}\left( \begin{matrix} 1 & r_{2,1} \\ r_{2,1} & 1 \end{matrix} \right)\left( \begin{matrix} e^{ik_{z,1}d_{1}} & 0 \\ 0 & e^{-ik_{z,1}d_{1}} \end{matrix} \right)\frac{1}{t_{10}}\left( \begin{matrix} 1 & r_{1,0} \\ r_{1,0} & 1 \end{matrix} \right)$ (7)

Or in a concise form:

$M=T_{n,n-1}P_{n-1}\cdots T_{2,1}P_{1}T_{1,0}$ (8)

Now, we must determine how the transmission coefficient $t_{\mathrm{total}}$ and the reflection coefficient $r_{\mathrm{total}}$ are related to the elements of the transfer matrix *M*. To do this, let us consider a plane wave incident from the left onto the multilayered structure. Then, up to a multiplicative factor, we can write: $A_{n}=t_{\mathrm{total}}$, $B_{n}=0$, $A_{0}=1$, and $B_{0}=r_{\mathrm{total}}$. As a result:

$r_{\mathrm{total}}=-\frac{M_{21}}{M_{22}}$ (9)

$t_{\mathrm{total}}=M_{11}-\frac{M_{12}M_{21}}{M_{22}}$ (10)

It is worth noting that for our calculations we used a bit more complex approach, described in the publication^[2]^, but similar principles are applied.

**Calculations for MoTe_2_ as δ-waveguides (monolayer waveguide)**


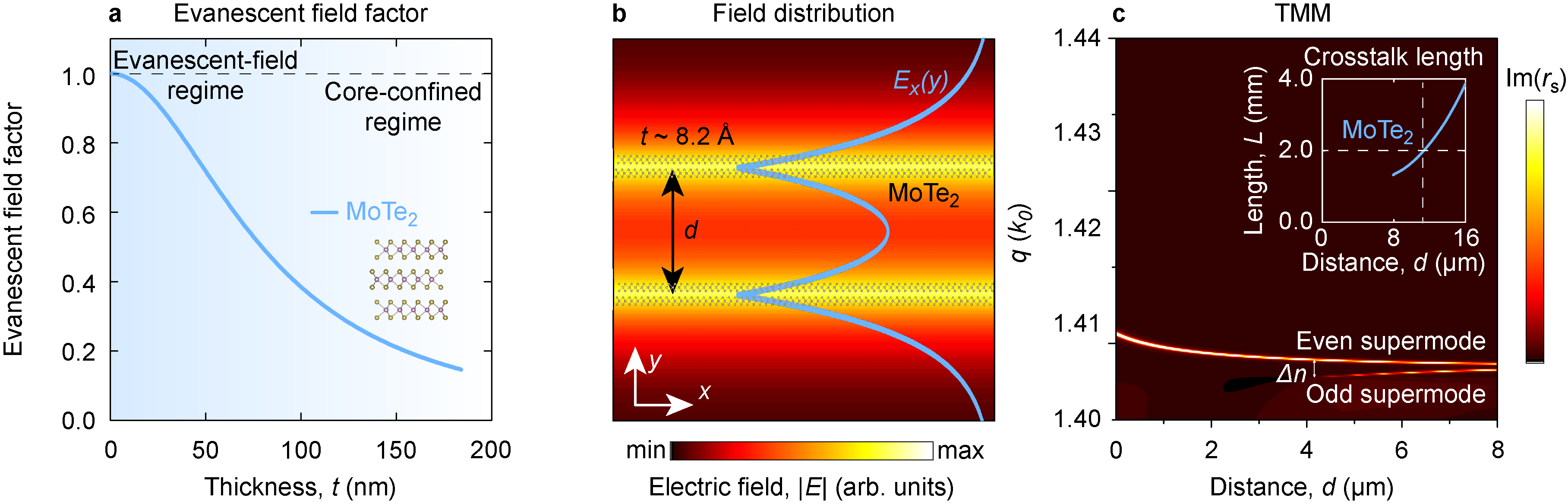


**Figure S5.** Crosstalk in MoTe_2_ monolayer waveguides. (a) Evanescent-field factor versus effective thickness. (b) Electric-field distribution |*E*| in a coupled MoTe_2_ waveguides. (c) Transfer-matrix calculation of even and odd supermodes and corresponding crosstalk length as a function of waveguide separation distance.

**Table S1.** Tabulated broadband optical constants of MoTe_2_.

| Wavelength, $\lambda$ (nm) | Refractive index, $n$ | Extinction coefficient, $k$ |
| --- | --- | --- |
| 260 | 2.22591 | 3.37870 |
| 261 | 2.34439 | 3.35914 |
| 262 | 2.46190 | 3.31356 |
| 263 | 2.56891 | 3.24152 |
| 264 | 2.65588 | 3.14732 |
| 265 | 2.71615 | 3.03948 |
| 266 | 2.74785 | 2.92837 |
| 267 | 2.75372 | 2.82325 |
| 268 | 2.73942 | 2.73045 |
| 269 | 2.71142 | 2.65297 |
| 270 | 2.67554 | 2.59117 |
| 271 | 2.63632 | 2.54384 |
| 272 | 2.59691 | 2.50898 |
| 273 | 2.55932 | 2.48442 |
| 274 | 2.52470 | 2.46813 |
| 275 | 2.49361 | 2.45834 |
| 276 | 2.46623 | 2.45358 |
| 277 | 2.44251 | 2.45267 |
| 278 | 2.42224 | 2.45465 |
| 279 | 2.40519 | 2.45881 |
| 280 | 2.39107 | 2.46458 |
| 281 | 2.37959 | 2.47150 |
| 282 | 2.37050 | 2.47924 |
| 283 | 2.36355 | 2.48755 |
| 284 | 2.35850 | 2.49621 |
| 285 | 2.35516 | 2.50508 |
| 286 | 2.35334 | 2.51404 |
| 287 | 2.35288 | 2.52299 |
| 288 | 2.35363 | 2.53187 |
| 289 | 2.35548 | 2.54063 |
| 290 | 2.35831 | 2.54922 |
| 291 | 2.36202 | 2.55762 |
| 292 | 2.36652 | 2.56580 |
| 293 | 2.37173 | 2.57375 |
| 294 | 2.37758 | 2.58146 |
| 295 | 2.38402 | 2.58891 |
| 296 | 2.39098 | 2.59611 |
| 297 | 2.39842 | 2.60304 |
| 298 | 2.40629 | 2.60972 |
| 299 | 2.41454 | 2.61613 |
| 300 | 2.42316 | 2.62228 |
| 301 | 2.43209 | 2.62817 |
| 302 | 2.44131 | 2.63381 |
| 303 | 2.45079 | 2.63919 |
| 304 | 2.46051 | 2.64432 |
| 305 | 2.47045 | 2.64921 |
| 306 | 2.48058 | 2.65385 |
| 307 | 2.49087 | 2.65826 |
| 308 | 2.50133 | 2.66243 |
| 309 | 2.51192 | 2.66637 |
| 310 | 2.52263 | 2.67008 |
| 311 | 2.53345 | 2.67357 |
| 312 | 2.54436 | 2.67685 |
| 313 | 2.55536 | 2.67991 |
| 314 | 2.56642 | 2.68276 |
| 315 | 2.57754 | 2.68541 |
| 316 | 2.58870 | 2.68785 |
| 317 | 2.59990 | 2.69011 |
| 318 | 2.61113 | 2.69217 |
| 319 | 2.62238 | 2.69405 |
| 320 | 2.63364 | 2.69574 |
| 321 | 2.64490 | 2.69726 |
| 322 | 2.65616 | 2.69860 |
| 323 | 2.66740 | 2.69978 |
| 324 | 2.67863 | 2.70080 |
| 325 | 2.68983 | 2.70165 |
| 326 | 2.70100 | 2.70236 |
| 327 | 2.71213 | 2.70291 |
| 328 | 2.72323 | 2.70332 |
| 329 | 2.73428 | 2.70360 |
| 330 | 2.74527 | 2.70373 |
| 331 | 2.75622 | 2.70374 |
| 332 | 2.76710 | 2.70362 |
| 333 | 2.77792 | 2.70338 |
| 334 | 2.78868 | 2.70303 |
| 335 | 2.79936 | 2.70257 |
| 336 | 2.80998 | 2.70200 |
| 337 | 2.82052 | 2.70133 |
| 338 | 2.83099 | 2.70056 |
| 339 | 2.84137 | 2.69970 |
| 340 | 2.85168 | 2.69876 |
| 341 | 2.86190 | 2.69773 |
| 342 | 2.87204 | 2.69662 |
| 343 | 2.88210 | 2.69545 |
| 344 | 2.89206 | 2.69420 |
| 345 | 2.90195 | 2.69289 |
| 346 | 2.91174 | 2.69151 |
| 347 | 2.92145 | 2.69009 |
| 348 | 2.93107 | 2.68861 |
| 349 | 2.94060 | 2.68708 |
| 350 | 2.95004 | 2.68551 |
| 351 | 2.95939 | 2.68390 |
| 352 | 2.96866 | 2.68225 |
| 353 | 2.97784 | 2.68058 |
| 354 | 2.98694 | 2.67887 |
| 355 | 2.99595 | 2.67714 |
| 356 | 3.00487 | 2.67539 |
| 357 | 3.01371 | 2.67363 |
| 358 | 3.02248 | 2.67184 |
| 359 | 3.03116 | 2.67005 |
| 360 | 3.03976 | 2.66825 |
| 361 | 3.04829 | 2.66645 |
| 362 | 3.05674 | 2.66464 |
| 363 | 3.06511 | 2.66283 |
| 364 | 3.07342 | 2.66103 |
| 365 | 3.08165 | 2.65923 |
| 366 | 3.08982 | 2.65744 |
| 367 | 3.09793 | 2.65566 |
| 368 | 3.10596 | 2.65389 |
| 369 | 3.11394 | 2.65214 |
| 370 | 3.12186 | 2.65040 |
| 371 | 3.12973 | 2.64868 |
| 372 | 3.13754 | 2.64698 |
| 373 | 3.14529 | 2.64530 |
| 374 | 3.15300 | 2.64364 |
| 375 | 3.16066 | 2.64201 |
| 376 | 3.16828 | 2.64040 |
| 377 | 3.17585 | 2.63882 |
| 378 | 3.18339 | 2.63726 |
| 379 | 3.19089 | 2.63573 |
| 380 | 3.19835 | 2.63423 |
| 381 | 3.20579 | 2.63276 |
| 382 | 3.21319 | 2.63131 |
| 383 | 3.22057 | 2.62990 |
| 384 | 3.22792 | 2.62852 |
| 385 | 3.23525 | 2.62716 |
| 386 | 3.24256 | 2.62584 |
| 387 | 3.24985 | 2.62454 |
| 388 | 3.25713 | 2.62328 |
| 389 | 3.26440 | 2.62204 |
| 390 | 3.27165 | 2.62084 |
| 391 | 3.27890 | 2.61966 |
| 392 | 3.28614 | 2.61851 |
| 393 | 3.29338 | 2.61739 |
| 394 | 3.30062 | 2.61630 |
| 395 | 3.30786 | 2.61524 |
| 396 | 3.31510 | 2.61420 |
| 397 | 3.32234 | 2.61318 |
| 398 | 3.32960 | 2.61219 |
| 399 | 3.33686 | 2.61122 |
| 400 | 3.34413 | 2.61028 |
| 401 | 3.35142 | 2.60936 |
| 402 | 3.35872 | 2.60845 |
| 403 | 3.36604 | 2.60757 |
| 404 | 3.37337 | 2.60670 |
| 405 | 3.38073 | 2.60585 |
| 406 | 3.38810 | 2.60502 |
| 407 | 3.39550 | 2.60420 |
| 408 | 3.40293 | 2.60339 |
| 409 | 3.41038 | 2.60259 |
| 410 | 3.41785 | 2.60180 |
| 411 | 3.42536 | 2.60102 |
| 412 | 3.43289 | 2.60024 |
| 413 | 3.44046 | 2.59947 |
| 414 | 3.44806 | 2.59871 |
| 415 | 3.45570 | 2.59794 |
| 416 | 3.46336 | 2.59718 |
| 417 | 3.47107 | 2.59641 |
| 418 | 3.47881 | 2.59564 |
| 419 | 3.48659 | 2.59486 |
| 420 | 3.49441 | 2.59407 |
| 421 | 3.50227 | 2.59328 |
| 422 | 3.51017 | 2.59247 |
| 423 | 3.51811 | 2.59165 |
| 424 | 3.52609 | 2.59082 |
| 425 | 3.53411 | 2.58997 |
| 426 | 3.54218 | 2.58910 |
| 427 | 3.55029 | 2.58821 |
| 428 | 3.55845 | 2.58729 |
| 429 | 3.56665 | 2.58635 |
| 430 | 3.57490 | 2.58539 |
| 431 | 3.58320 | 2.58439 |
| 432 | 3.59154 | 2.58336 |
| 433 | 3.59992 | 2.58230 |
| 434 | 3.60836 | 2.58120 |
| 435 | 3.61684 | 2.58007 |
| 436 | 3.62537 | 2.57889 |
| 437 | 3.63394 | 2.57768 |
| 438 | 3.64257 | 2.57641 |
| 439 | 3.65124 | 2.57511 |
| 440 | 3.65996 | 2.57375 |
| 441 | 3.66872 | 2.57234 |
| 442 | 3.67754 | 2.57087 |
| 443 | 3.68640 | 2.56935 |
| 444 | 3.69531 | 2.56777 |
| 445 | 3.70426 | 2.56613 |
| 446 | 3.71327 | 2.56443 |
| 447 | 3.72232 | 2.56265 |
| 448 | 3.73141 | 2.56081 |
| 449 | 3.74055 | 2.55890 |
| 450 | 3.74973 | 2.55692 |
| 451 | 3.75896 | 2.55485 |
| 452 | 3.76824 | 2.55271 |
| 453 | 3.77755 | 2.55049 |
| 454 | 3.78691 | 2.54818 |
| 455 | 3.79631 | 2.54578 |
| 456 | 3.80575 | 2.54329 |
| 457 | 3.81523 | 2.54071 |
| 458 | 3.82474 | 2.53803 |
| 459 | 3.83430 | 2.53525 |
| 460 | 3.84389 | 2.53237 |
| 461 | 3.85351 | 2.52938 |
| 462 | 3.86317 | 2.52628 |
| 463 | 3.87286 | 2.52307 |
| 464 | 3.88258 | 2.51975 |
| 465 | 3.89233 | 2.51630 |
| 466 | 3.90210 | 2.51273 |
| 467 | 3.91190 | 2.50904 |
| 468 | 3.92172 | 2.50521 |
| 469 | 3.93156 | 2.50126 |
| 470 | 3.94142 | 2.49716 |
| 471 | 3.95129 | 2.49292 |
| 472 | 3.96118 | 2.48854 |
| 473 | 3.97107 | 2.48401 |
| 474 | 3.98097 | 2.47933 |
| 475 | 3.99087 | 2.47449 |
| 476 | 4.00077 | 2.46948 |
| 477 | 4.01067 | 2.46431 |
| 478 | 4.02055 | 2.45898 |
| 479 | 4.03042 | 2.45346 |
| 480 | 4.04027 | 2.44777 |
| 481 | 4.05010 | 2.44189 |
| 482 | 4.05990 | 2.43583 |
| 483 | 4.06966 | 2.42957 |
| 484 | 4.07938 | 2.42312 |
| 485 | 4.08905 | 2.41646 |
| 486 | 4.09866 | 2.40960 |
| 487 | 4.10821 | 2.40253 |
| 488 | 4.11768 | 2.39524 |
| 489 | 4.12708 | 2.38773 |
| 490 | 4.13638 | 2.38000 |
| 491 | 4.14557 | 2.37204 |
| 492 | 4.15466 | 2.36385 |
| 493 | 4.16362 | 2.35543 |
| 494 | 4.17244 | 2.34677 |
| 495 | 4.18110 | 2.33787 |
| 496 | 4.18960 | 2.32873 |
| 497 | 4.19792 | 2.31934 |
| 498 | 4.20604 | 2.30971 |
| 499 | 4.21394 | 2.29984 |
| 500 | 4.22161 | 2.28973 |
| 501 | 4.22903 | 2.27938 |
| 502 | 4.23617 | 2.26881 |
| 503 | 4.24302 | 2.25800 |
| 504 | 4.24955 | 2.24698 |
| 505 | 4.25574 | 2.23575 |
| 506 | 4.26156 | 2.22432 |
| 507 | 4.26701 | 2.21272 |
| 508 | 4.27204 | 2.20095 |
| 509 | 4.27664 | 2.18905 |
| 510 | 4.28079 | 2.17703 |
| 511 | 4.28446 | 2.16493 |
| 512 | 4.28764 | 2.15277 |
| 513 | 4.29030 | 2.14059 |
| 514 | 4.29244 | 2.12844 |
| 515 | 4.29404 | 2.11636 |
| 516 | 4.29510 | 2.10438 |
| 517 | 4.29561 | 2.09257 |
| 518 | 4.29559 | 2.08098 |
| 519 | 4.29503 | 2.06967 |
| 520 | 4.29397 | 2.05868 |
| 521 | 4.29243 | 2.04807 |
| 522 | 4.29044 | 2.03791 |
| 523 | 4.28807 | 2.02824 |
| 524 | 4.28536 | 2.01912 |
| 525 | 4.28238 | 2.01058 |
| 526 | 4.27922 | 2.00267 |
| 527 | 4.27596 | 1.99540 |
| 528 | 4.27271 | 1.98879 |
| 529 | 4.26960 | 1.98285 |
| 530 | 4.26676 | 1.97754 |
| 531 | 4.26441 | 1.97281 |
| 532 | 4.26286 | 1.96833 |
| 533 | 4.26177 | 1.96358 |
| 534 | 4.26086 | 1.95865 |
| 535 | 4.26005 | 1.95359 |
| 536 | 4.25927 | 1.94843 |
| 537 | 4.25848 | 1.94319 |
| 538 | 4.25766 | 1.93791 |
| 539 | 4.25678 | 1.93259 |
| 540 | 4.25583 | 1.92725 |
| 541 | 4.25478 | 1.92192 |
| 542 | 4.25363 | 1.91659 |
| 543 | 4.25237 | 1.91130 |
| 544 | 4.25099 | 1.90604 |
| 545 | 4.24949 | 1.90084 |
| 546 | 4.24785 | 1.89571 |
| 547 | 4.24607 | 1.89066 |
| 548 | 4.24416 | 1.88570 |
| 549 | 4.24211 | 1.88085 |
| 550 | 4.23992 | 1.87611 |
| 551 | 4.23759 | 1.87151 |
| 552 | 4.23512 | 1.86705 |
| 553 | 4.23252 | 1.86275 |
| 554 | 4.22978 | 1.85861 |
| 555 | 4.22692 | 1.85466 |
| 556 | 4.22393 | 1.85089 |
| 557 | 4.22082 | 1.84733 |
| 558 | 4.21761 | 1.84398 |
| 559 | 4.21429 | 1.84086 |
| 560 | 4.21088 | 1.83798 |
| 561 | 4.20739 | 1.83534 |
| 562 | 4.20382 | 1.83296 |
| 563 | 4.20019 | 1.83085 |
| 564 | 4.19651 | 1.82902 |
| 565 | 4.19280 | 1.82747 |
| 566 | 4.18906 | 1.82621 |
| 567 | 4.18532 | 1.82525 |
| 568 | 4.18159 | 1.82461 |
| 569 | 4.17789 | 1.82427 |
| 570 | 4.17423 | 1.82425 |
| 571 | 4.17064 | 1.82456 |
| 572 | 4.16713 | 1.82519 |
| 573 | 4.16373 | 1.82616 |
| 574 | 4.16046 | 1.82745 |
| 575 | 4.15735 | 1.82907 |
| 576 | 4.15441 | 1.83101 |
| 577 | 4.15168 | 1.83328 |
| 578 | 4.14919 | 1.83587 |
| 579 | 4.14696 | 1.83878 |
| 580 | 4.14504 | 1.84198 |
| 581 | 4.14347 | 1.84547 |
| 582 | 4.14228 | 1.84924 |
| 583 | 4.14156 | 1.85325 |
| 584 | 4.14142 | 1.85745 |
| 585 | 4.14193 | 1.86154 |
| 586 | 4.14284 | 1.86543 |
| 587 | 4.14405 | 1.86915 |
| 588 | 4.14552 | 1.87272 |
| 589 | 4.14723 | 1.87615 |
| 590 | 4.14913 | 1.87945 |
| 591 | 4.15123 | 1.88264 |
| 592 | 4.15350 | 1.88571 |
| 593 | 4.15593 | 1.88867 |
| 594 | 4.15850 | 1.89153 |
| 595 | 4.16122 | 1.89428 |
| 596 | 4.16407 | 1.89694 |
| 597 | 4.16704 | 1.89951 |
| 598 | 4.17012 | 1.90198 |
| 599 | 4.17332 | 1.90436 |
| 600 | 4.17662 | 1.90666 |
| 601 | 4.18002 | 1.90887 |
| 602 | 4.18352 | 1.91100 |
| 603 | 4.18710 | 1.91304 |
| 604 | 4.19077 | 1.91500 |
| 605 | 4.19453 | 1.91689 |
| 606 | 4.19836 | 1.91869 |
| 607 | 4.20227 | 1.92042 |
| 608 | 4.20625 | 1.92207 |
| 609 | 4.21029 | 1.92364 |
| 610 | 4.21441 | 1.92514 |
| 611 | 4.21859 | 1.92657 |
| 612 | 4.22283 | 1.92793 |
| 613 | 4.22713 | 1.92921 |
| 614 | 4.23148 | 1.93043 |
| 615 | 4.23589 | 1.93157 |
| 616 | 4.24035 | 1.93264 |
| 617 | 4.24486 | 1.93365 |
| 618 | 4.24941 | 1.93458 |
| 619 | 4.25402 | 1.93545 |
| 620 | 4.25866 | 1.93626 |
| 621 | 4.26335 | 1.93699 |
| 622 | 4.26808 | 1.93766 |
| 623 | 4.27285 | 1.93827 |
| 624 | 4.27766 | 1.93881 |
| 625 | 4.28250 | 1.93928 |
| 626 | 4.28738 | 1.93970 |
| 627 | 4.29229 | 1.94004 |
| 628 | 4.29723 | 1.94033 |
| 629 | 4.30220 | 1.94056 |
| 630 | 4.30719 | 1.94072 |
| 631 | 4.31222 | 1.94082 |
| 632 | 4.31727 | 1.94086 |
| 633 | 4.32235 | 1.94084 |
| 634 | 4.32745 | 1.94076 |
| 635 | 4.33257 | 1.94062 |
| 636 | 4.33772 | 1.94042 |
| 637 | 4.34288 | 1.94016 |
| 638 | 4.34807 | 1.93984 |
| 639 | 4.35327 | 1.93947 |
| 640 | 4.35849 | 1.93904 |
| 641 | 4.36372 | 1.93855 |
| 642 | 4.36897 | 1.93800 |
| 643 | 4.37424 | 1.93740 |
| 644 | 4.37951 | 1.93674 |
| 645 | 4.38480 | 1.93602 |
| 646 | 4.39011 | 1.93525 |
| 647 | 4.39542 | 1.93442 |
| 648 | 4.40074 | 1.93354 |
| 649 | 4.40607 | 1.93261 |
| 650 | 4.41141 | 1.93162 |
| 651 | 4.41675 | 1.93058 |
| 652 | 4.42210 | 1.92948 |
| 653 | 4.42746 | 1.92833 |
| 654 | 4.43282 | 1.92713 |
| 655 | 4.43818 | 1.92587 |
| 656 | 4.44355 | 1.92457 |
| 657 | 4.44892 | 1.92321 |
| 658 | 4.45430 | 1.92180 |
| 659 | 4.45967 | 1.92034 |
| 660 | 4.46505 | 1.91883 |
| 661 | 4.47042 | 1.91727 |
| 662 | 4.47580 | 1.91566 |
| 663 | 4.48117 | 1.91400 |
| 664 | 4.48654 | 1.91229 |
| 665 | 4.49191 | 1.91053 |
| 666 | 4.49728 | 1.90872 |
| 667 | 4.50264 | 1.90687 |
| 668 | 4.50800 | 1.90496 |
| 669 | 4.51336 | 1.90301 |
| 670 | 4.51871 | 1.90101 |
| 671 | 4.52405 | 1.89896 |
| 672 | 4.52939 | 1.89686 |
| 673 | 4.53472 | 1.89471 |
| 674 | 4.54004 | 1.89251 |
| 675 | 4.54535 | 1.89026 |
| 676 | 4.55065 | 1.88797 |
| 677 | 4.55594 | 1.88563 |
| 678 | 4.56122 | 1.88325 |
| 679 | 4.56648 | 1.88082 |
| 680 | 4.57174 | 1.87834 |
| 681 | 4.57697 | 1.87582 |
| 682 | 4.58220 | 1.87326 |
| 683 | 4.58741 | 1.87065 |
| 684 | 4.59260 | 1.86800 |
| 685 | 4.59778 | 1.86530 |
| 686 | 4.60294 | 1.86256 |
| 687 | 4.60809 | 1.85978 |
| 688 | 4.61321 | 1.85696 |
| 689 | 4.61832 | 1.85410 |
| 690 | 4.62342 | 1.85119 |
| 691 | 4.62849 | 1.84825 |
| 692 | 4.63354 | 1.84526 |
| 693 | 4.63858 | 1.84224 |
| 694 | 4.64359 | 1.83917 |
| 695 | 4.64859 | 1.83607 |
| 696 | 4.65356 | 1.83293 |
| 697 | 4.65851 | 1.82975 |
| 698 | 4.66344 | 1.82654 |
| 699 | 4.66835 | 1.82328 |
| 700 | 4.67323 | 1.81999 |
| 701 | 4.67810 | 1.81667 |
| 702 | 4.68294 | 1.81330 |
| 703 | 4.68775 | 1.80991 |
| 704 | 4.69254 | 1.80647 |
| 705 | 4.69731 | 1.80301 |
| 706 | 4.70205 | 1.79951 |
| 707 | 4.70677 | 1.79597 |
| 708 | 4.71147 | 1.79240 |
| 709 | 4.71613 | 1.78880 |
| 710 | 4.72077 | 1.78517 |
| 711 | 4.72539 | 1.78150 |
| 712 | 4.72998 | 1.77780 |
| 713 | 4.73454 | 1.77407 |
| 714 | 4.73908 | 1.77032 |
| 715 | 4.74358 | 1.76653 |
| 716 | 4.74806 | 1.76271 |
| 717 | 4.75252 | 1.75886 |
| 718 | 4.75694 | 1.75498 |
| 719 | 4.76134 | 1.75107 |
| 720 | 4.76570 | 1.74713 |
| 721 | 4.77004 | 1.74317 |
| 722 | 4.77435 | 1.73918 |
| 723 | 4.77863 | 1.73516 |
| 724 | 4.78288 | 1.73112 |
| 725 | 4.78710 | 1.72705 |
| 726 | 4.79130 | 1.72295 |
| 727 | 4.79546 | 1.71883 |
| 728 | 4.79959 | 1.71468 |
| 729 | 4.80369 | 1.71051 |
| 730 | 4.80776 | 1.70632 |
| 731 | 4.81179 | 1.70210 |
| 732 | 4.81580 | 1.69785 |
| 733 | 4.81978 | 1.69359 |
| 734 | 4.82372 | 1.68930 |
| 735 | 4.82764 | 1.68499 |
| 736 | 4.83152 | 1.68066 |
| 737 | 4.83537 | 1.67630 |
| 738 | 4.83919 | 1.67193 |
| 739 | 4.84297 | 1.66754 |
| 740 | 4.84672 | 1.66312 |
| 741 | 4.85045 | 1.65869 |
| 742 | 4.85413 | 1.65423 |
| 743 | 4.85779 | 1.64976 |
| 744 | 4.86141 | 1.64527 |
| 745 | 4.86500 | 1.64076 |
| 746 | 4.86856 | 1.63623 |
| 747 | 4.87208 | 1.63169 |
| 748 | 4.87558 | 1.62713 |
| 749 | 4.87903 | 1.62255 |
| 750 | 4.88246 | 1.61795 |
| 751 | 4.88585 | 1.61334 |
| 752 | 4.88921 | 1.60872 |
| 753 | 4.89253 | 1.60408 |
| 754 | 4.89582 | 1.59942 |
| 755 | 4.89908 | 1.59475 |
| 756 | 4.90230 | 1.59007 |
| 757 | 4.90549 | 1.58537 |
| 758 | 4.90865 | 1.58066 |
| 759 | 4.91177 | 1.57594 |
| 760 | 4.91486 | 1.57121 |
| 761 | 4.91791 | 1.56646 |
| 762 | 4.92093 | 1.56170 |
| 763 | 4.92392 | 1.55693 |
| 764 | 4.92687 | 1.55215 |
| 765 | 4.92979 | 1.54736 |
| 766 | 4.93267 | 1.54256 |
| 767 | 4.93552 | 1.53774 |
| 768 | 4.93834 | 1.53292 |
| 769 | 4.94112 | 1.52809 |
| 770 | 4.94386 | 1.52325 |
| 771 | 4.94658 | 1.51841 |
| 772 | 4.94926 | 1.51355 |
| 773 | 4.95190 | 1.50869 |
| 774 | 4.95451 | 1.50382 |
| 775 | 4.95709 | 1.49894 |
| 776 | 4.95963 | 1.49405 |
| 777 | 4.96214 | 1.48916 |
| 778 | 4.96462 | 1.48427 |
| 779 | 4.96706 | 1.47936 |
| 780 | 4.96947 | 1.47445 |
| 781 | 4.97184 | 1.46954 |
| 782 | 4.97418 | 1.46462 |
| 783 | 4.97648 | 1.45970 |
| 784 | 4.97876 | 1.45477 |
| 785 | 4.98099 | 1.44984 |
| 786 | 4.98320 | 1.44491 |
| 787 | 4.98537 | 1.43997 |
| 788 | 4.98751 | 1.43503 |
| 789 | 4.98961 | 1.43009 |
| 790 | 4.99168 | 1.42515 |
| 791 | 4.99372 | 1.42020 |
| 792 | 4.99572 | 1.41525 |
| 793 | 4.99769 | 1.41030 |
| 794 | 4.99963 | 1.40535 |
| 795 | 5.00153 | 1.40040 |
| 796 | 5.00340 | 1.39545 |
| 797 | 5.00524 | 1.39050 |
| 798 | 5.00704 | 1.38555 |
| 799 | 5.00882 | 1.38060 |
| 800 | 5.01056 | 1.37565 |
| 801 | 5.01226 | 1.37070 |
| 802 | 5.01394 | 1.36575 |
| 803 | 5.01558 | 1.36081 |
| 804 | 5.01719 | 1.35587 |
| 805 | 5.01877 | 1.35092 |
| 806 | 5.02031 | 1.34598 |
| 807 | 5.02182 | 1.34105 |
| 808 | 5.02330 | 1.33611 |
| 809 | 5.02475 | 1.33118 |
| 810 | 5.02617 | 1.32626 |
| 811 | 5.02756 | 1.32134 |
| 812 | 5.02891 | 1.31642 |
| 813 | 5.03023 | 1.31150 |
| 814 | 5.03152 | 1.30659 |
| 815 | 5.03278 | 1.30169 |
| 816 | 5.03401 | 1.29679 |
| 817 | 5.03521 | 1.29189 |
| 818 | 5.03638 | 1.28700 |
| 819 | 5.03751 | 1.28212 |
| 820 | 5.03862 | 1.27724 |
| 821 | 5.03969 | 1.27237 |
| 822 | 5.04074 | 1.26751 |
| 823 | 5.04175 | 1.26265 |
| 824 | 5.04274 | 1.25780 |
| 825 | 5.04369 | 1.25296 |
| 826 | 5.04462 | 1.24812 |
| 827 | 5.04551 | 1.24329 |
| 828 | 5.04638 | 1.23847 |
| 829 | 5.04721 | 1.23366 |
| 830 | 5.04802 | 1.22886 |
| 831 | 5.04879 | 1.22406 |
| 832 | 5.04954 | 1.21927 |
| 833 | 5.05026 | 1.21450 |
| 834 | 5.05095 | 1.20973 |
| 835 | 5.05161 | 1.20497 |
| 836 | 5.05224 | 1.20022 |
| 837 | 5.05285 | 1.19548 |
| 838 | 5.05342 | 1.19075 |
| 839 | 5.05397 | 1.18603 |
| 840 | 5.05449 | 1.18132 |
| 841 | 5.05498 | 1.17663 |
| 842 | 5.05544 | 1.17194 |
| 843 | 5.05588 | 1.16726 |
| 844 | 5.05629 | 1.16260 |
| 845 | 5.05667 | 1.15795 |
| 846 | 5.05702 | 1.15330 |
| 847 | 5.05735 | 1.14868 |
| 848 | 5.05765 | 1.14406 |
| 849 | 5.05793 | 1.13945 |
| 850 | 5.05817 | 1.13486 |
| 851 | 5.05839 | 1.13028 |
| 852 | 5.05859 | 1.12571 |
| 853 | 5.05876 | 1.12116 |
| 854 | 5.05890 | 1.11661 |
| 855 | 5.05901 | 1.11209 |
| 856 | 5.05911 | 1.10757 |
| 857 | 5.05917 | 1.10307 |
| 858 | 5.05921 | 1.09858 |
| 859 | 5.05923 | 1.09411 |
| 860 | 5.05922 | 1.08965 |
| 861 | 5.05918 | 1.08520 |
| 862 | 5.05912 | 1.08077 |
| 863 | 5.05904 | 1.07636 |
| 864 | 5.05893 | 1.07196 |
| 865 | 5.05879 | 1.06757 |
| 866 | 5.05864 | 1.06320 |
| 867 | 5.05845 | 1.05884 |
| 868 | 5.05825 | 1.05450 |
| 869 | 5.05802 | 1.05018 |
| 870 | 5.05777 | 1.04587 |
| 871 | 5.05749 | 1.04158 |
| 872 | 5.05719 | 1.03730 |
| 873 | 5.05687 | 1.03304 |
| 874 | 5.05652 | 1.02879 |
| 875 | 5.05616 | 1.02456 |
| 876 | 5.05577 | 1.02035 |
| 877 | 5.05535 | 1.01616 |
| 878 | 5.05492 | 1.01198 |
| 879 | 5.05446 | 1.00782 |
| 880 | 5.05398 | 1.00368 |
| 881 | 5.05348 | 0.99955 |
| 882 | 5.05296 | 0.99544 |
| 883 | 5.05241 | 0.99135 |
| 884 | 5.05185 | 0.98728 |
| 885 | 5.05126 | 0.98322 |
| 886 | 5.05065 | 0.97919 |
| 887 | 5.05002 | 0.97517 |
| 888 | 5.04938 | 0.97117 |
| 889 | 5.04871 | 0.96719 |
| 890 | 5.04802 | 0.96322 |
| 891 | 5.04731 | 0.95928 |
| 892 | 5.04658 | 0.95535 |
| 893 | 5.04583 | 0.95145 |
| 894 | 5.04506 | 0.94756 |
| 895 | 5.04427 | 0.94369 |
| 896 | 5.04346 | 0.93984 |
| 897 | 5.04263 | 0.93601 |
| 898 | 5.04178 | 0.93220 |
| 899 | 5.04092 | 0.92841 |
| 900 | 5.04003 | 0.92465 |
| 901 | 5.03913 | 0.92090 |
| 902 | 5.03821 | 0.91717 |
| 903 | 5.03727 | 0.91346 |
| 904 | 5.03631 | 0.90977 |
| 905 | 5.03533 | 0.90610 |
| 906 | 5.03434 | 0.90245 |
| 907 | 5.03333 | 0.89883 |
| 908 | 5.03230 | 0.89522 |
| 909 | 5.03125 | 0.89164 |
| 910 | 5.03019 | 0.88807 |
| 911 | 5.02911 | 0.88453 |
| 912 | 5.02802 | 0.88101 |
| 913 | 5.02690 | 0.87751 |
| 914 | 5.02577 | 0.87404 |
| 915 | 5.02463 | 0.87058 |
| 916 | 5.02347 | 0.86715 |
| 917 | 5.02229 | 0.86374 |
| 918 | 5.02110 | 0.86035 |
| 919 | 5.01989 | 0.85698 |
| 920 | 5.01866 | 0.85364 |
| 921 | 5.01742 | 0.85032 |
| 922 | 5.01617 | 0.84702 |
| 923 | 5.01490 | 0.84374 |
| 924 | 5.01362 | 0.84049 |
| 925 | 5.01232 | 0.83726 |
| 926 | 5.01101 | 0.83405 |
| 927 | 5.00968 | 0.83087 |
| 928 | 5.00834 | 0.82771 |
| 929 | 5.00699 | 0.82458 |
| 930 | 5.00562 | 0.82146 |
| 931 | 5.00424 | 0.81838 |
| 932 | 5.00285 | 0.81531 |
| 933 | 5.00144 | 0.81227 |
| 934 | 5.00002 | 0.80926 |
| 935 | 4.99859 | 0.80626 |
| 936 | 4.99715 | 0.80330 |
| 937 | 4.99569 | 0.80035 |
| 938 | 4.99422 | 0.79744 |
| 939 | 4.99274 | 0.79455 |
| 940 | 4.99125 | 0.79168 |
| 941 | 4.98975 | 0.78884 |
| 942 | 4.98824 | 0.78602 |
| 943 | 4.98671 | 0.78323 |
| 944 | 4.98518 | 0.78046 |
| 945 | 4.98363 | 0.77772 |
| 946 | 4.98208 | 0.77501 |
| 947 | 4.98051 | 0.77232 |
| 948 | 4.97894 | 0.76966 |
| 949 | 4.97735 | 0.76702 |
| 950 | 4.97576 | 0.76441 |
| 951 | 4.97416 | 0.76183 |
| 952 | 4.97255 | 0.75927 |
| 953 | 4.97093 | 0.75674 |
| 954 | 4.96930 | 0.75424 |
| 955 | 4.96767 | 0.75177 |
| 956 | 4.96603 | 0.74932 |
| 957 | 4.96438 | 0.74690 |
| 958 | 4.96272 | 0.74450 |
| 959 | 4.96106 | 0.74214 |
| 960 | 4.95940 | 0.73980 |
| 961 | 4.95772 | 0.73749 |
| 962 | 4.95605 | 0.73521 |
| 963 | 4.95437 | 0.73295 |
| 964 | 4.95269 | 0.73072 |
| 965 | 4.95100 | 0.72851 |
| 966 | 4.94930 | 0.72633 |
| 967 | 4.94761 | 0.72417 |
| 968 | 4.94590 | 0.72204 |
| 969 | 4.94420 | 0.71994 |
| 970 | 4.94248 | 0.71787 |
| 971 | 4.94077 | 0.71582 |
| 972 | 4.93905 | 0.71380 |
| 973 | 4.93732 | 0.71180 |
| 974 | 4.93559 | 0.70983 |
| 975 | 4.93386 | 0.70789 |
| 976 | 4.93212 | 0.70598 |
| 977 | 4.93038 | 0.70409 |
| 978 | 4.92864 | 0.70223 |
| 979 | 4.92689 | 0.70040 |
| 980 | 4.92514 | 0.69860 |
| 981 | 4.92339 | 0.69683 |
| 982 | 4.92164 | 0.69508 |
| 983 | 4.91988 | 0.69337 |
| 984 | 4.91812 | 0.69168 |
| 985 | 4.91636 | 0.69002 |
| 986 | 4.91460 | 0.68839 |
| 987 | 4.91284 | 0.68679 |
| 988 | 4.91108 | 0.68522 |
| 989 | 4.90932 | 0.68368 |
| 990 | 4.90756 | 0.68217 |
| 991 | 4.90579 | 0.68069 |
| 992 | 4.90403 | 0.67924 |
| 993 | 4.90227 | 0.67782 |
| 994 | 4.90051 | 0.67642 |
| 995 | 4.89876 | 0.67506 |
| 996 | 4.89700 | 0.67374 |
| 997 | 4.89525 | 0.67244 |
| 998 | 4.89349 | 0.67117 |
| 999 | 4.89175 | 0.66993 |
| 1000 | 4.89000 | 0.66873 |
| 1010 | 4.87283 | 0.65843 |
| 1020 | 4.85647 | 0.65142 |
| 1030 | 4.84145 | 0.64778 |
| 1040 | 4.82842 | 0.64753 |
| 1050 | 4.81814 | 0.65056 |
| 1060 | 4.81147 | 0.65655 |
| 1070 | 4.80932 | 0.66494 |
| 1080 | 4.81261 | 0.67481 |
| 1090 | 4.82205 | 0.68483 |
| 1100 | 4.83804 | 0.69331 |
| 1110 | 4.86038 | 0.69827 |
| 1120 | 4.88814 | 0.69769 |
| 1130 | 4.91962 | 0.68988 |
| 1140 | 4.95249 | 0.67380 |
| 1150 | 4.98418 | 0.64937 |
| 1160 | 5.01225 | 0.61742 |
| 1170 | 5.03486 | 0.57956 |
| 1180 | 5.05094 | 0.53780 |
| 1190 | 5.06018 | 0.49417 |
| 1200 | 5.06292 | 0.45045 |
| 1210 | 5.05992 | 0.40801 |
| 1220 | 5.05211 | 0.36779 |
| 1230 | 5.04046 | 0.33036 |
| 1240 | 5.02587 | 0.29599 |
| 1250 | 5.00913 | 0.26472 |
| 1260 | 4.99089 | 0.23648 |
| 1270 | 4.97167 | 0.21110 |
| 1280 | 4.95189 | 0.18838 |
| 1290 | 4.93184 | 0.16809 |
| 1300 | 4.91178 | 0.15001 |
| 1310 | 4.89189 | 0.13395 |
| 1320 | 4.87230 | 0.11971 |
| 1330 | 4.85312 | 0.10711 |
| 1340 | 4.83442 | 0.09601 |
| 1350 | 4.81627 | 0.08627 |
| 1360 | 4.79872 | 0.07776 |
| 1370 | 4.78182 | 0.07039 |
| 1380 | 4.76563 | 0.06405 |
| 1390 | 4.75028 | 0.05864 |
| 1400 | 4.73584 | 0.05385 |
| 1410 | 4.72213 | 0.04949 |
| 1420 | 4.70905 | 0.04554 |
| 1430 | 4.69655 | 0.04193 |
| 1440 | 4.68458 | 0.03864 |
| 1450 | 4.67310 | 0.03563 |
| 1460 | 4.66208 | 0.03287 |
| 1470 | 4.65150 | 0.03034 |
| 1480 | 4.64132 | 0.02802 |
| 1490 | 4.63152 | 0.02588 |
| 1500 | 4.62209 | 0.02392 |
| 1510 | 4.61299 | 0.02210 |
| 1520 | 4.60422 | 0.02043 |
| 1530 | 4.59575 | 0.01888 |
| 1540 | 4.58758 | 0.01745 |
| 1550 | 4.57968 | 0.01613 |
| 1560 | 4.57203 | 0.01490 |
| 1570 | 4.56464 | 0.01376 |
| 1580 | 4.55749 | 0.01270 |
| 1590 | 4.55056 | 0.01172 |
| 1600 | 4.54384 | 0.01080 |
| 1610 | 4.53733 | 0.00996 |
| 1620 | 4.53102 | 0.00917 |
| 1630 | 4.52489 | 0.00843 |
| 1640 | 4.51894 | 0.00775 |
| 1650 | 4.51317 | 0.00711 |
| 1660 | 4.50755 | 0.00652 |
| 1670 | 4.50210 | 0.00597 |
| 1680 | 4.49680 | 0.00545 |
| 1690 | 4.49164 | 0.00497 |
| 1700 | 4.48662 | 0.00453 |
| 1710 | 4.48173 | 0.00411 |
| 1720 | 4.47698 | 0.00373 |
| 1730 | 4.47235 | 0.00337 |
| 1740 | 4.46783 | 0.00304 |
| 1750 | 4.46344 | 0.00273 |
| 1760 | 4.45915 | 0.00245 |
| 1770 | 4.45497 | 0.00219 |
| 1780 | 4.45089 | 0.00194 |
| 1790 | 4.44692 | 0.00172 |
| 1800 | 4.44304 | 0.00151 |
| 1810 | 4.43925 | 0.00132 |
| 1820 | 4.43555 | 0.00115 |
| 1830 | 4.43194 | 0.00099 |
| 1840 | 4.42842 | 0.00085 |
| 1850 | 4.42498 | 0.00072 |
| 1860 | 4.42161 | 0.00060 |
| 1870 | 4.41832 | 0.00049 |
| 1880 | 4.41511 | 0.00040 |
| 1890 | 4.41197 | 0.00032 |
| 1900 | 4.40890 | 0.00025 |
| 1910 | 4.40590 | 0.00018 |
| 1920 | 4.40297 | 0.00013 |
| 1930 | 4.40010 | 0.00009 |
| 1940 | 4.39730 | 0.00006 |
| 1950 | 4.39456 | 0.00003 |
| 1960 | 4.39187 | 0.00001 |
| 1970 | 4.38925 | 0 |
| 1980 | 4.38669 | 0.00000 |
| 1990 | 4.38420 | 0.00000 |
| 2000 | 4.38175 | 0.00000 |
| 2010 | 4.37936 | 0.00000 |
| 2020 | 4.37702 | 0.00000 |
| 2030 | 4.37473 | 0.00000 |
| 2040 | 4.37249 | 0.00000 |
| 2050 | 4.37029 | 0.00000 |
| 2060 | 4.36814 | 0.00000 |
| 2070 | 4.36603 | 0.00000 |
| 2080 | 4.36396 | 0.00000 |
| 2090 | 4.36193 | 0.00000 |
| 2100 | 4.35994 | 0.00000 |
| 2110 | 4.35798 | 0.00000 |
| 2120 | 4.35606 | 0.00000 |
| 2130 | 4.35418 | 0.00000 |
| 2140 | 4.35233 | 0.00000 |
| 2150 | 4.35052 | 0.00000 |
| 2160 | 4.34873 | 0.00000 |
| 2170 | 4.34698 | 0.00000 |
| 2180 | 4.34526 | 0.00000 |
| 2190 | 4.34357 | 0.00000 |
| 2200 | 4.34191 | 0.00000 |
| 2210 | 4.34028 | 0.00000 |
| 2220 | 4.33868 | 0.00000 |
| 2230 | 4.33710 | 0.00000 |
| 2240 | 4.33555 | 0.00000 |
| 2250 | 4.33403 | 0.00000 |
| 2260 | 4.33253 | 0.00000 |
| 2270 | 4.33105 | 0.00000 |
| 2280 | 4.32960 | 0.00000 |
| 2290 | 4.32817 | 0.00000 |
| 2300 | 4.32677 | 0.00000 |
| 2310 | 4.32539 | 0.00000 |
| 2320 | 4.32403 | 0.00000 |
| 2330 | 4.32269 | 0.00000 |
| 2340 | 4.32137 | 0.00000 |
| 2350 | 4.32007 | 0.00000 |
| 2360 | 4.31879 | 0.00000 |
| 2370 | 4.31754 | 0.00000 |
| 2380 | 4.31630 | 0.00000 |
| 2390 | 4.31508 | 0.00000 |
| 2400 | 4.31388 | 0.00000 |
| 2410 | 4.31269 | 0.00000 |
| 2420 | 4.31152 | 0.00000 |
| 2430 | 4.31038 | 0.00000 |
| 2440 | 4.30924 | 0.00000 |
| 2450 | 4.30813 | 0.00000 |
| 2460 | 4.30703 | 0.00000 |
| 2470 | 4.30594 | 0.00000 |
| 2480 | 4.30487 | 0.00000 |
| 2490 | 4.30382 | 0.00000 |
| 2500 | 4.30278 | 0.00000 |
| 2510 | 4.30176 | 0.00000 |
| 2520 | 4.30075 | 0.00000 |
| 2530 | 4.29975 | 0.00000 |
| 2540 | 4.29877 | 0.00000 |
| 2550 | 4.29780 | 0.00000 |
| 2560 | 4.29685 | 0.00000 |
| 2570 | 4.29590 | 0.00000 |
| 2580 | 4.29497 | 0.00000 |
| 2590 | 4.29406 | 0.00000 |
| 2600 | 4.29315 | 0.00000 |
| 2610 | 4.29226 | 0.00000 |
| 2620 | 4.29138 | 0.00000 |
| 2630 | 4.29051 | 0.00000 |
| 2640 | 4.28965 | 0.00000 |
| 2650 | 4.28880 | 0.00000 |
| 2660 | 4.28797 | 0.00000 |
| 2670 | 4.28714 | 0.00000 |
| 2680 | 4.28633 | 0.00000 |
| 2690 | 4.28552 | 0.00000 |
| 2700 | 4.28473 | 0.00000 |
| 2710 | 4.28395 | 0.00000 |
| 2720 | 4.28317 | 0.00000 |
| 2730 | 4.28241 | 0.00000 |
| 2740 | 4.28165 | 0.00000 |
| 2750 | 4.28091 | 0.00000 |
| 2760 | 4.28017 | 0.00000 |
| 2770 | 4.27944 | 0.00000 |
| 2780 | 4.27872 | 0.00000 |
| 2790 | 4.27801 | 0.00000 |
| 2800 | 4.27731 | 0.00000 |
| 2810 | 4.27662 | 0.00000 |
| 2820 | 4.27594 | 0.00000 |
| 2830 | 4.27526 | 0.00000 |
| 2840 | 4.27459 | 0.00000 |
| 2850 | 4.27393 | 0.00000 |
| 2860 | 4.27328 | 0.00000 |
| 2870 | 4.27263 | 0.00000 |
| 2880 | 4.27200 | 0.00000 |
| 2890 | 4.27137 | 0.00000 |
| 2900 | 4.27074 | 0.00000 |
| 2910 | 4.27013 | 0.00000 |
| 2920 | 4.26952 | 0.00000 |
| 2930 | 4.26892 | 0.00000 |
| 2940 | 4.26832 | 0.00000 |
| 2950 | 4.26773 | 0.00000 |
| 2960 | 4.26715 | 0.00000 |
| 2970 | 4.26658 | 0.00000 |
| 2980 | 4.26601 | 0.00000 |
| 2990 | 4.26545 | 0.00000 |
| 3000 | 4.26489 | 0.00000 |
| 3010 | 4.26434 | 0.00000 |
| 3020 | 4.26380 | 0.00000 |
| 3030 | 4.26326 | 0.00000 |
| 3040 | 4.26273 | 0.00000 |
| 3050 | 4.26220 | 0.00000 |
| 3060 | 4.26168 | 0.00000 |
| 3070 | 4.26116 | 0.00000 |
| 3080 | 4.26066 | 0.00000 |
| 3090 | 4.26015 | 0.00000 |
| 3100 | 4.25965 | 0.00000 |
| 3110 | 4.25916 | 0.00000 |
| 3120 | 4.25867 | 0.00000 |
| 3130 | 4.25819 | 0.00000 |
| 3140 | 4.25771 | 0.00000 |
| 3150 | 4.25724 | 0.00000 |
| 3160 | 4.25677 | 0.00000 |
| 3170 | 4.25631 | 0.00000 |
| 3180 | 4.25585 | 0.00000 |
| 3190 | 4.25539 | 0.00000 |
| 3200 | 4.25494 | 0.00000 |
| 3210 | 4.25450 | 0.00000 |
| 3220 | 4.25406 | 0.00000 |
| 3230 | 4.25362 | 0.00000 |
| 3240 | 4.25319 | 0.00000 |
| 3250 | 4.25277 | 0.00000 |
| 3260 | 4.25234 | 0.00000 |
| 3270 | 4.25192 | 0.00000 |
| 3280 | 4.25151 | 0.00000 |
| 3290 | 4.25110 | 0.00000 |
| 3300 | 4.25069 | 0.00000 |
| 3310 | 4.25029 | 0.00000 |
| 3320 | 4.24989 | 0.00000 |
| 3330 | 4.24950 | 0.00000 |
| 3340 | 4.24911 | 0.00000 |
| 3350 | 4.24872 | 0.00000 |
| 3360 | 4.24834 | 0.00000 |
| 3370 | 4.24796 | 0.00000 |
| 3380 | 4.24758 | 0.00000 |
| 3390 | 4.24721 | 0.00000 |
| 3400 | 4.24684 | 0.00000 |
| 3410 | 4.24648 | 0.00000 |
| 3420 | 4.24612 | 0.00000 |
| 3430 | 4.24576 | 0.00000 |
| 3440 | 4.24540 | 0.00000 |
| 3450 | 4.24505 | 0.00000 |
| 3460 | 4.24470 | 0.00000 |
| 3470 | 4.24436 | 0.00000 |
| 3480 | 4.24402 | 0.00000 |
| 3490 | 4.24368 | 0.00000 |
| 3500 | 4.24334 | 0.00000 |
| 3510 | 4.24301 | 0.00000 |
| 3520 | 4.24268 | 0.00000 |
| 3530 | 4.24236 | 0.00000 |
| 3540 | 4.24203 | 0.00000 |
| 3550 | 4.24171 | 0.00000 |
| 3560 | 4.24139 | 0.00000 |
| 3570 | 4.24108 | 0.00000 |
| 3580 | 4.24077 | 0.00000 |
| 3590 | 4.24046 | 0.00000 |
| 3600 | 4.24015 | 0.00000 |
| 3610 | 4.23985 | 0.00000 |
| 3620 | 4.23955 | 0.00000 |
| 3630 | 4.23925 | 0.00000 |
| 3640 | 4.23895 | 0.00000 |
| 3650 | 4.23866 | 0.00000 |
| 3660 | 4.23837 | 0.00000 |
| 3670 | 4.23808 | 0.00000 |
| 3680 | 4.23780 | 0.00000 |
| 3690 | 4.23751 | 0.00000 |
| 3700 | 4.23723 | 0.00000 |
| 3710 | 4.23695 | 0.00000 |
| 3720 | 4.23668 | 0.00000 |
| 3730 | 4.23641 | 0.00000 |
| 3740 | 4.23613 | 0.00000 |
| 3750 | 4.23587 | 0.00000 |
| 3760 | 4.23560 | 0.00000 |
| 3770 | 4.23533 | 0.00000 |
| 3780 | 4.23507 | 0.00000 |
| 3790 | 4.23481 | 0.00000 |
| 3800 | 4.23455 | 0.00000 |
| 3810 | 4.23430 | 0.00000 |
| 3820 | 4.23405 | 0.00000 |
| 3830 | 4.23379 | 0.00000 |
| 3840 | 4.23355 | 0.00000 |
| 3850 | 4.23330 | 0.00000 |
| 3860 | 4.23305 | 0.00000 |
| 3870 | 4.23281 | 0.00000 |
| 3880 | 4.23257 | 0.00000 |
| 3890 | 4.23233 | 0.00000 |
| 3900 | 4.23209 | 0.00000 |
| 3910 | 4.23186 | 0.00000 |
| 3920 | 4.23162 | 0.00000 |
| 3930 | 4.23139 | 0.00000 |
| 3940 | 4.23116 | 0.00000 |
| 3950 | 4.23093 | 0.00000 |
| 3960 | 4.23071 | 0.00000 |
| 3970 | 4.23048 | 0.00000 |
| 3980 | 4.23026 | 0.00000 |
| 3990 | 4.23004 | 0.00000 |
| 4000 | 4.22982 | 0.00000 |
| 4010 | 4.22961 | 0.00000 |
| 4020 | 4.22939 | 0.00000 |
| 4030 | 4.22918 | 0.00000 |
| 4040 | 4.22896 | 0.00000 |
| 4050 | 4.22875 | 0.00000 |
| 4060 | 4.22854 | 0.00000 |
| 4070 | 4.22834 | 0.00000 |
| 4080 | 4.22813 | 0.00000 |
| 4090 | 4.22793 | 0.00000 |
| 4100 | 4.22773 | 0.00000 |
| 4110 | 4.22752 | 0.00000 |
| 4120 | 4.22733 | 0.00000 |
| 4130 | 4.22713 | 0.00000 |
| 4140 | 4.22693 | 0.00000 |
| 4150 | 4.22674 | 0.00000 |
| 4160 | 4.22654 | 0.00000 |
| 4170 | 4.22635 | 0.00000 |
| 4180 | 4.22616 | 0.00000 |
| 4190 | 4.22597 | 0.00000 |
| 4200 | 4.22578 | 0.00000 |
| 4210 | 4.22560 | 0.00000 |
| 4220 | 4.22541 | 0.00000 |
| 4230 | 4.22523 | 0.00000 |
| 4240 | 4.22505 | 0.00000 |
| 4250 | 4.22487 | 0.00000 |
| 4260 | 4.22469 | 0.00000 |
| 4270 | 4.22451 | 0.00000 |
| 4280 | 4.22433 | 0.00000 |
| 4290 | 4.22416 | 0.00000 |
| 4300 | 4.22398 | 0.00000 |
| 4310 | 4.22381 | 0.00000 |
| 4320 | 4.22364 | 0.00000 |
| 4330 | 4.22347 | 0.00000 |
| 4340 | 4.22330 | 0.00000 |
| 4350 | 4.22313 | 0.00000 |
| 4360 | 4.22296 | 0.00000 |
| 4370 | 4.22280 | 0.00000 |
| 4380 | 4.22263 | 0.00000 |
| 4390 | 4.22247 | 0.00000 |
| 4400 | 4.22231 | 0.00000 |
| 4410 | 4.22215 | 0.00000 |
| 4420 | 4.22199 | 0.00000 |
| 4430 | 4.22183 | 0.00000 |
| 4440 | 4.22167 | 0.00000 |
| 4450 | 4.22151 | 0.00000 |
| 4460 | 4.22136 | 0.00000 |
| 4470 | 4.22120 | 0.00000 |
| 4480 | 4.22105 | 0.00000 |
| 4490 | 4.22090 | 0.00000 |
| 4500 | 4.22075 | 0.00000 |
| 4510 | 4.22060 | 0.00000 |
| 4520 | 4.22045 | 0.00000 |
| 4530 | 4.22030 | 0.00000 |
| 4540 | 4.22015 | 0.00000 |
| 4550 | 4.22001 | 0.00000 |
| 4560 | 4.21986 | 0.00000 |
| 4570 | 4.21972 | 0.00000 |
| 4580 | 4.21958 | 0.00000 |
| 4590 | 4.21943 | 0.00000 |
| 4600 | 4.21929 | 0.00000 |
| 4610 | 4.21915 | 0.00000 |
| 4620 | 4.21901 | 0.00000 |
| 4630 | 4.21888 | 0.00000 |
| 4640 | 4.21874 | 0.00000 |
| 4650 | 4.21860 | 0.00000 |
| 4660 | 4.21847 | 0.00000 |
| 4670 | 4.21833 | 0.00000 |
| 4680 | 4.21820 | 0.00000 |
| 4690 | 4.21807 | 0.00000 |
| 4700 | 4.21793 | 0.00000 |
| 4710 | 4.21780 | 0.00000 |
| 4720 | 4.21767 | 0.00000 |
| 4730 | 4.21754 | 0.00000 |
| 4740 | 4.21742 | 0.00000 |
| 4750 | 4.21729 | 0.00000 |
| 4760 | 4.21716 | 0.00000 |
| 4770 | 4.21704 | 0.00000 |
| 4780 | 4.21691 | 0.00000 |
| 4790 | 4.21679 | 0.00000 |
| 4800 | 4.21666 | 0.00000 |
| 4810 | 4.21654 | 0.00000 |
| 4820 | 4.21642 | 0.00000 |
| 4830 | 4.21630 | 0.00000 |
| 4840 | 4.21618 | 0.00000 |
| 4850 | 4.21606 | 0.00000 |
| 4860 | 4.21594 | 0.00000 |
| 4870 | 4.21582 | 0.00000 |
| 4880 | 4.21570 | 0.00000 |
| 4890 | 4.21559 | 0.00000 |
| 4900 | 4.21547 | 0.00000 |
| 4910 | 4.21535 | 0.00000 |
| 4920 | 4.21524 | 0.00000 |
| 4930 | 4.21513 | 0.00000 |
| 4940 | 4.21501 | 0.00000 |
| 4950 | 4.21490 | 0.00000 |
| 4960 | 4.21479 | 0.00000 |
| 4970 | 4.21468 | 0.00000 |
| 4980 | 4.21457 | 0.00000 |
| 4990 | 4.21446 | 0.00000 |
| 5000 | 4.21435 | 0.00000 |

**References**

[1] A. Slavich, G. Ermolaev, I. Zavidovskiy, D. Grudinin, M. Tatmyshevskiy, A. Toksumakov, A. Syuy, A. Vyshnevyy, D. Yakubovsky, S. Novikov, D. Ghazaryan, A. Arsenin, V. Volkov, *Bull. Russ. Acad. Sci. Phys.* **2025**, DOI 10.1134/S1062873824709978.

[2] N. C. Passler, A. Paarmann, *J. Opt. Soc. Am. B* **2017**, *34*, 2128.
